# Effectiveness of oseltamivir in hospitalised obstetric patients with COVID-19: a retrospective cohort study using a Brazilian national database

**DOI:** 10.1186/s12879-025-12327-x

**Published:** 2025-12-20

**Authors:** Angela Burvill, Ana Cristina Simões e Silva, Char Leung, Li Su

**Affiliations:** 1 School of Clinical Medicine, University of Cambridge, Box 111, Cambridge Biomedical Campus, Cambridge, CB2 0SP UK; 2 Present Address: Sir Charles Gairdner Hospital, Hospital Avenue, Nedlands, 6009 Australia; 3 School of Medicine, Federal University of Minas Gerais, Av. Prof. Alfredo Balena, Belo Horizonte, 30130-100 Brazil; 4 Department of Population Health Sciences, University of Leicester, George Davies Centre, University Road, Leicester, LE1 7RH UK; 5 MRC Biostatistics Unit, School of Clinical Medicine, University of Cambridge, Box 111, Cambridge Biomedical Campus, Cambridge, CB2 0SP UK

**Keywords:** COVID-19, SARS-CoV-2, Oseltamivir, Neuraminidase inhibitor, Pregnant, Obstetric

## Abstract

**Background:**

Effective, safe, and accessible antivirals are needed to treat pregnant/postpartum (obstetric) patients with COVID-19, a group at high risk for severe disease. Newer antivirals (nirmatrelvir/ritonavir, remdesivir) are expensive and less accessible in low- and middle-income countries, including Brazil. Oseltamivir, a cheap, widely available, pregnancy-safe anti-influenza medication, was used off-label for COVID-19 in Brazil. The primary outcome was comparing in-hospital all-cause mortality in hospitalised obstetric patients with COVID-19 treated with oseltamivir versus no antivirals. Secondary outcomes were comparing the risk of progression to severe disease (ICU admission or death, whichever occurred first) and hospital discharge.

**Methods:**

In this retrospective matched cohort study using Brazil’s national surveillance database (SIVEP-Gripe), we identified hospitalised obstetric patients with PCR-confirmed COVID-19 between February 2020 and October 2023. Patients first receiving oseltamivir on day zero of admission and admitted within seven days of symptom onset were matched 1:1 using propensity scores to patients receiving no antivirals at all.

**Results:**

After matching, 445 oseltamivir recipients and 445 controls were included, of whom 79.5% and 80.0%, respectively, were admitted in 2020, and 65.8% and 67.9% had non-severe COVID-19 on admission (SpO2 > 94%). Oseltamivir use was associated with a lower risk of in-hospital death (hazard ratio [HR] 0.77; [95% CI 0.51–1.17], *p* = 0.22; absolute risk reduction [ARR] 3.9%) and progression to severe disease (HR 0.83 [95% CI [0.67–1.01], *p* = 0.07; ARR 4.5%) compared with no antivirals, though these associations were not statistically significant overall. Oseltamivir recipients were less likely to be discharged on days 0–2 (HR 0.68 [0.52–0.90]) but more likely to be discharged on or after day 3 (HR 1.30 [1.10–1.54]) than controls. Significant associations between oseltamivir use and lower in-hospital mortality were observed in two subgroups: patients admitted in 2020 (HR 0.54 [95% CI 0.32–0.93], *p* = 0.03) and patients with non-severe COVID-19 (SpO2 > 94%) on admission (HR 0.33 [95% CI 0.12–0.89], *p* = 0.03).

**Conclusions:**

In obstetric patients with COVID-19, oseltamivir used within seven days of symptom onset was associated with non-statistically significant lower risk of in-hospital death and progression to severe disease, compared with no antivirals. Results of this study do not support use of oseltamivir to treat COVID-19 in this population.

**Supplementary Information:**

The online version contains supplementary material available at 10.1186/s12879-025-12327-x.

## Introduction

Effective, safe and accessible antivirals are needed to treat pregnant and postpartum (henceforth obstetric) patients with coronavirus disease (COVID-19), a population at high risk of severe disease [1].

Current World Health Organisation (WHO) guidelines recommend considering nirmatrelvir/ritonavir (Paxlovid®) or remdesivir for pregnant patients, regardless of vaccination status [1]. However, these medications are expensive [2], have not been formally tested in obstetric patients [1] and remdesivir is given intravenously which is challenging for outpatients. These limitations reduce their accessibility, especially in low- and middle-income countries.

Oseltamivir, an anti-influenza medication, was used frequently off-label to treat COVID-19 at the start of the pandemic, especially in Brazil [3, 4]. In national treatment guidelines for obstetric patients published by the Brazilian Ministry of Health in 2020 and 2021 [5, 6], it was recommended that pregnant patients with ‘fever’ or ‘cough’ or ‘myalgia’ or arthralgia’ or ‘headache’ and whose symptoms commenced less than 48 hours prior be treated with a five-day course of oseltamivir. Despite a global shift away from using oseltamivir for COVID-19 after 2021 [7], its accessibility still makes it a candidate treatment in resource-limited settings.

Oseltamivir is the first-line medication to treat influenza in pregnant patients [8]. It is cheap, widely available and safe in pregnancy [9] . Oseltamivir is effective at reducing mortality from influenza when given up to seven days from symptom onset, with the greatest benefits when given within 48 hours [10]. This provided a plausible rationale for its off-label use for COVID-19.

While observational studies in non-obstetric populations suggested that oseltamivir might be associated with lower COVID-19 mortality compared with no antivirals [11, 12], obstetric patients have been largely excluded from both clinical trials and observational studies of repurposed medications to treat COVID-19 [13]. Therefore, the effectiveness of oseltamivir in the obstetric population remains unclear.

To address this gap, we compared in-hospital all-cause mortality (as the primary outcome) in hospitalised obstetric patients with COVID-19 treated with oseltamivir versus no antivirals. Secondary outcomes were comparing the risk of progression to severe disease (ICU admission or death, whichever occurred first) and hospital discharge. We used data from the Influenza Epidemiological Surveillance Information System (Sistema de Informação de Vigilância Epidemiológica da Gripe, SIVEP-Gripe) in Brazil [14]. Our aim was to determine if oseltamivir could represent a low-cost antiviral to complement treatment of COVID-19 in this group.

The SIVEP-Gripe is Brazil’s national database for the surveillance of COVID-19 and has been used in previous studies to investigate maternal morbidity and mortality from COVID-19 [15]. Notification to the SIVEP-Gripe was mandatory for all COVID-19 hospitalisations (public or private). Sadly, Brazil experienced one of the highest maternal mortality rates from COVID-19 [16]. Further, the SIVEP-Gripe coded for oseltamivir and other influenza antivirals but did not code for Paxlovid® or remdesivir, which were unavailable in Brazil until 2022 [17]. This makes “no antivirals at all” the only feasible comparator group for investigating oseltamivir within this database.

## Methods

### Study design

**Fig. 1:**
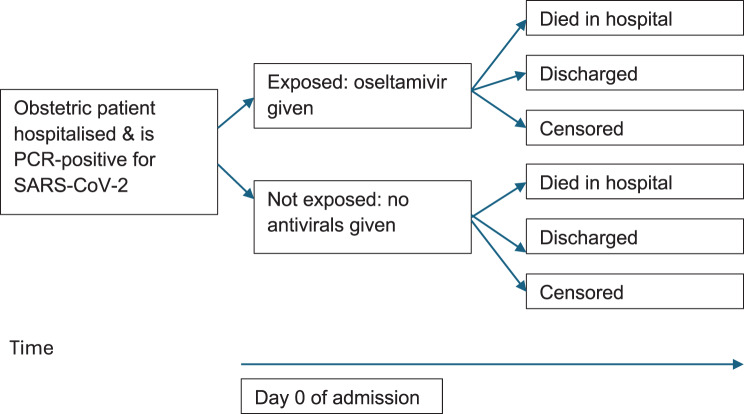
Study design – retrospective cohort study.Those with missing data for the clinical endpoint were coded as censored

This was a retrospective cohort study using the Brazilian national database SIVEP-Gripe. Figure 1 illustrates the study design and direction of enquiry. We included hospitalised obstetric patients with PCR-confirmed SARS-CoV-2 infection admitted from February 2020 (the index case of SARS-CoV-2 in Brazil) to 2 October 2023 (the date of data extraction cut-off) to maximise sample size. This study followed the STROBE guidelines for reporting cohort studies (Supplementary A) [18]. As it used publicly available, anonymised data, ethical approval was therefore not required for this study in neither Brazil nor the United Kingdom.

### Data sources and study population

We analysed anonymised individual patient data from the publicly available SIVEP-Gripe database. This SIVEP-Gripe was the primary source of data on COVID-19-related hospital admissions in Brazil and is described elsewhere [19]. Basic demographic and medical data were systematically recorded in a standardised electronic form. Data were verified by the medical practitioner at the point of care [19]. Region, ethnicity and symptoms were self-reported. Laboratory data were not available. The type of antiviral used and date of first use were reported but the information on the dose and duration of therapy was not provided. Authors did not have access to information that could identify individual participants during or after data collection.

Patients were eligible for inclusion in this study if they met all of the following criteria at hospital admission: (i) PCR-positive for SARS-CoV-2; (ii) pregnant or postpartum (within six weeks of delivery) and female; (iii) aged 15 to 45 inclusive; (iv) PCR negative or missing for influenza; (v) date of hospital admission was available, because this was the index date for follow-up; (vi) admitted to hospital within seven days of symptom onset, because COVID-19 specific antivirals are usually prescribed within seven days [20]; (vii) oseltamivir or no antivirals were administered; (viii) for patients in the oseltamivir group, known date of antiviral administration and oseltamivir was first administered on the date of hospital admission.

We excluded patients who had missing data for age; used an antiviral therapy other than oseltamivir; and patients who first used oseltamivir before or after the day zero of hospital admission. There was no predetermined sample size.

### Treatment exposure and follow-up

Treatment exposure was defined as those who received oseltamivir (as the only antiviral) on day zero of admission. Controls were selected, using propensity score matching, from hospitalised obstetric patients with COVID-19 who did not receive oseltamivir nor another antiviral therapy.Supplementary B summarises the Brazilian national guidelines in 2020 and 2021 for prescribing oseltamivir to pregnant patients with COVID-19, which stated that oseltamivir should be administered within 48 hours of onset of flu-like symptoms [5, 6]. We used seven days because there were insufficient cases in the dataset when restricting patients admitted within 48 hours.

Treatment exposure and the index date were both defined as day zero of hospital admission. By excluding patients who first used oseltamivir before or after the first day of hospital admission, we avoided immortal time bias (the time between treatment initiation and follow-up) [21]. That is, those who first used oseltamivir before day zero of admission were guaranteed to be alive between treatment initiation and hospital admission, which could introduce bias. Initiation after admission may have involved confounding by indication and we didn’t have detailed data to examine the disease progression between admission and treatment initiation.

Patients were followed from the index date until they developed an outcome (in-hospital death or discharge) or were censored (Fig. 1). Those with missing data for the clinical endpoint (*n* = 61/890) were coded as censored. We defined the censoring time as the maximum length of hospital stay in the matched cohort (114 days) or until October 2023 (the date of data extraction), whichever was earlier. For those with missing data for date of discharge (*n* = 15/890), we imputed time to discharge based on the median values of time of those who were in the same calendar time period (2020 or 2021 onwards), treatment group and time from symptom onset category ( < 7 days or ≥ 7 days), as these were the main baseline factors known to likely affect time to hospital discharge.Supplementary C gives further details.

### Outcomes

The primary outcome was in-hospital death (all-causes). We defined ‘ICU admission or in-hospital death, whichever occurred firstly’ as a secondary composite outcome, which indicated progression to severe disease. The SIVEP-Gripe had data on ICU admission. WHO guidelines stated that ICU admission can be investigated as an outcome, if more granular data on interventions (e.g. non-invasive ventilation) were not available [22]. We defined another secondary outcome as hospital-discharge (alive). WHO guidelines stated that in-hospital mortality (all-cause) and duration of hospital stay were important outcomes in COVID-19 clinical research[22].

### Baseline covariates

Patients’ baseline covariate data were collected from the SIVEP-Gripe. We chose covariates that may be associated with treatment assignment or study outcomes [23]: age; public or private hospital; region of residence and ethnicity, which reflected inequity of access to healthcare in Brazil [24]; pregnancy stage at admission, which can affect risk of severe COVID-19 [25]; normal oxygen saturation (SpO2 > 94%) on admission (yes/no), with SpO2 > 94% indicating non-severe COVID-19 [1, 22]; self-reported symptoms and pre-existing comorbidities (all of those reported in the database); vaccination to COVID-19 or influenza (prior to hospital admission), which can affect risk of severe COVID-19 [26]; time from symptom onset to admission, because this affects decisions to prescribe antiviral therapies [5, 6]; and calendar period of admission, to reflect changes in SARS-CoV-2 variants and COVID-19 treatment over time in Brazil. Ethnicity had a separate category for ‘missing or not reported’ data. Data were complete for the other covariates. Additional details on each baseline covariate are provided in Supplementary D.

### Statistical analysis

We used propensity score matching to optimise the balance between treatment groups with respect to baseline characteristics. (Supplementary C gives the model specification) [27]. Propensity score matching is a method to mimic randomisation in a randomised controlled trial (RCT). [27] We estimated the propensity scores of oseltamivir usage using a logistic regression model with the aforementioned baseline covariates. Covariates with more than two categories were treated as categorical variables. There were first order interaction terms between ‘time from symptom onset’ and fever, cough and headache, as these factors determined treatment assignment (Supplementary B).

Using estimated propensity scores, oseltamivir recipients were matched with controls by applying 1:1 nearest neighbour matching without replacement and with a calliper width of 0.05. We chose the 1:1 matching because this ratio provides an interpretable comparison and it is the standard ratio in clinical trial designs. Using only one untreated subject for each treated subject will tend to minimise bias [23, 28]. Standardised mean differences (SMD) < 0.1 indicated the baseline covariates were well balanced [27]. We plotted the distribution of SMDs before and after matching.

Cumulative incidence functions were presented as non-parametric summary of outcome distributions. The absolute risk reduction (ARR) and number needed to treat (NNT) generated from the cumulative incidence functions were for description only.

Cox regression models were used to estimate cause-specific hazard ratios (HR) with 95% confidence intervals (CI). This investigated the association of oseltamivir, versus no antivirals at all, on the risk of in-hospital death while considering competing risks, and separately for hospital discharge and composite outcome. Unadjusted HRs with robust standard errors were estimated. In the model for in-hospital death or composite outcome, hospital discharge was a censoring variable. In the model for hospital discharge, in-hospital death was a censoring variable. For each Cox regression model, we assessed the proportional hazards assumption of the treatment groups by using the global Schoenfeld’s residual test for violation of the proportional hazards assumption. Where the proportional hazards assumption was violated, we split the follow-up time into intervals. We repeated analyses using Fine-Gray sub-distribution HRs with robust standard errors for the outcomes, to complement treatment effect estimates using Cox models [29]. Supplementary C gives details.

Subgroup analyses were performed for the following patient groups: (1) study period (before 1 January 2021 or after), to reflect changes in SARS-CoV-2 variates and in treatment guidelines and disruptions to the Brazilian healthcare system over time [5, 6, 30]; (2) SpO2 > 94%, because this was the only objective indicator of non-severe versus severe COVID-19 before treatment assignment [1, 22]; (3) third trimester patients, who may have been admitted as a precaution; (4) postpartum, who had higher risk of mortality [31]; and (5) vaccinated for SARS-CoV-2 or not, because there was lower risk of death in vaccinated patients [32]. For each subgroup analysis, we used the same propensity score model as the main analysis but restricted analyses to only those in the subgroup.

Over the follow-up period, the proportion of patients in each clinical state were compared between oseltamivir recipients and their matched controls.Supplementary E shows possible transitions between clinical states.

The causal interpretation of treatment effects using propensity score matching relies on the standard assumptions of exchangeability, positivity and consistency. Exchangeability (no unmeasured confounding) was addressed by including a comprehensive set of baseline covariates, including demographics, clinical, pregnancy-related, symptoms-based, comorbidities, vaccination status, and illness-severity factors (including SpO₂). These factors reflected oseltamivir prescribing guidelines in the Brazilian guidelines (Supplementary B). Positivity was supported by the substantial overlap in estimated propensity score distributions between treatment groups in the matched cohort (Supplementary F) and excellent covariate balance after matching (SMD < 0.1; Fig. 3 and Table 1). Consistency was assumed, meaning that the recorded prescriptions of oseltamivir corresponded to the same intervention across all treated patients.

**Table 1:** Baseline characteristics of oseltamivir recipients and control groups, before and after matching

Characteristics	Unmatched			Matched		
	Oseltamivir recipient (*n* = 463)	Control (*n* = 6595)	SMD	Oseltamivir recipient (*n* = 445)	Control (*n* = 445)	SMD
Age, y (median) [IQR]	29.5 [24.6, 35.0]	29.4 [24.4, 34.8]	< 0.01	29.5 [24.8, 35.1]	29.4 [23.8, 34.3]	0.06
Public hospital, %	272 (58.7%)	3143 (47.7%)	0.23 †	256 (57.5%)	262 (58.9%)	0.03
Pregnant or postpartum						
First trimester	36 (7.8%)	462 (7.0%)	0.03	35 (7.9%)	32 (7.2%)	0.03
Second trimester	105 (22.7%)	1124 (17.0%)	0.13 †	102 (22.9%)	102 (22.9%)	0.00
Third trimester	223 (48.2%)	3200 (48.5%)	< 0.01	214 (48.1%)	210 (47.2%)	0.02
Postpartum	99 (21.4%)	1809 (27.4%)	0.15 †	94 (21.1%)	101 (22.7%)	0.04
Region of Brazil						
Centre West	43 (9.3%)	705 (10.7%)	0.05	43 (9.7%)	44 (9.9%)	0.01
North	78 (16.8%)	301 (4.6%)	0.33 †	63 (14.2%)	64 (14.4%)	0.01
Northeast	78 (16.8%)	1147 (17.4%)	0.01	78 (17.5%)	87 (19.6%)	0.05
South	29 (6.3%)	1472 (22.3%)	0.66 †	29 (6.5%)	26 (5.8%)	0.03
Southeast	235 (50.8%)	2970 (45.0%)	0.11 †	232 (52.1%)	224 (50.3%)	0.04
Self-reported ethnicity						
African	28 (6.0%)	385 (5.8%)	< 0.01	26 (5.8%)	26 (5.8%)	0.00
Asian	2 (0.4%)	47 (0.7%)	0.04	2 (0.4%)	1 (0.2%)	0.03
Caucasian	136 (29.4%)	2752 (41.7%)	0.27 †	136 (30.6%)	119 (26.7%)	0.08
Indigenous	1 (0.2%)	17 (0.3%)	< 0.01	1 (0.2%)	2 (0.4%)	0.05
Mixed	224 (48.4%)	2477 (37.6%)	0.22 †	70 (15.7%)	74 (16.6%)	0.06
Missing or not reported	72 (15.6%)	917 (13.9%)	0.04	210 (47.2%)	223 (50.1%)	0.02
Signs						
SpO2 > 94% on admission	306 (66.1%)	4736 (71.8%)	0.12 †	293 (65.8%)	298 (67.0%)	0.02
Symptoms						
Fever	328 (70.8%)	3123 (47.4%)	0.52 †	310 (69.7%)	302 (67.9%)	0.04
Cough	379 (81.9%)	4035 (61.2%)	0.54 †	361 (81.1%)	364 (81.8%)	0.02
Sore throat	121 (26.1%)	1336 (20.3%)	0.11 †	112 (25.2%)	116 (26.1%)	0.02
Dyspnoea	288 (62.2%)	2850 (43.2%)	0.39 †	271 (60.9%)	267 (60.0%)	0.02
Respiratory discomfort	225 (48.6%)	2349 (35.6%)	0.26 †	211 (47.2%)	216 (48.5%)	0.02
Nasal congestion	49 (10.6%)	849 (12.9%)	0.07	49 (11.0%)	45 (10.1%)	0.03
Loss of smell (anosmia)	34 (7.3%)	592 (9.0%)	0.06	34 (7.6%)	31 (7.0%)	0.03
Loss of taste (ageusia)	29 (6.3%)	552 (8.4%)	0.09	29 (6.5%)	31 (7.0%)	0.02
Fatigue	38 (8.2%)	978 (14.8%)	0.24 †	38 (8.5%)	43 (9.7%)	0.04
Headache	69 (14.9%)	907 (13.8%)	0.03	69 (15.5%)	68 (15.3%)	0.01
Diarrhoea	39 (8.4%)	450 (6.8%)	0.06	38 (8.5%)	32 (7.2%)	0.05
Vomit	46 (9.9%)	541 (8.2%)	0.06	43 (9.7%)	49 (11.0%)	0.04
Abdominal pain	14 (3.0%)	400 (6.1%)	0.18 †	14 (3.1%)	13 (2.9%)	0.01
Nausea	4 (0.9%)	69 (1.0%)	0.02	4 (0.9%)	5 (1.1%)	0.02
Comorbidities						
Cardiovascular disease	39 (8.4%)	366 (5.5%)	0.11 †	37 (8.3%)	33 (7.4%)	0.03
Diabetes	38 (8.2%)	457 (6.9%)	0.05	38 (8.5%)	31 (7.0%)	0.06
Obesity	26 (5.6%)	363 (5.5%)	< 0.01	26 (5.8%)	25 (5.6%)	0.01
Asthma	20 (4.3%)	231 (3.5%)	0.04	20 (4.5%)	19 (4.3%)	0.01
Immunocompromised	8 (1.7%)	63 (1.0%)	0.06	6 (1.3%)	9 (2.0%)	0.05
Liver disease	1 (0.2%)	18 (0.3%)	0.01	1 (0.2%)	0 (0.0%)	0.05
Neurological disease	6 (1.3%)	47 (0.7%)	0.05	6 (1.3%)	5 (1.1%)	0.02
Renal disease	1 (0.2%)	38 (0.6%)	0.08	1 (0.2%)	2 (0.4%)	0.05
Down syndrome	0 (0.0%)	9 (0.1%)	0.04	0 (0.0%)	0 (0.0%	0.00
Vaccinations						
SARS-CoV-2	21 (4.5%)	1572 (23.8%)	0.93 †	21 (4.7%)	20 (4.5%)	0.01
Influenza	97 (21.0%)	1014 (15.4%)	0.14 †	96 (21.6%)	78 (17.5%)	0.10
Days from symptom onset to hospital admission						
0	45 (9.7%)	1491 (22.6%)	0.43 †	45 (10.1%)	42 (9.4%)	0.02
1	56 (12.1%)	878 (13.3%)	0.04	56 (12.6%)	64 (14.4%)	0.05
2	64 (13.8%)	830 (12.6%)	0.04	61 (13.7%)	58 (13.0%)	0.02
3	107 (23.1%)	958 (14.5%)	0.20 †	97 (21.8%)	93 (20.9%)	0.02
4	78 (16.8%)	838 (12.7%)	0.11 †	75 (16.9%)	79 (17.8%)	0.02
5	66 (14.3%)	768 (11.6%)	0.07	64 (14.4%)	61 (13.7%)	0.02
6	47 (10.2%)	832 (12.6%)	0.08	47 (10.6%)	48 (10.8%)	< 0.01
Quarter of admission						
Jan-Mar 2020	7 (1.5%)	25 (0.4%)	0.09	7 (1.6%)	7 (1.6%)	0.00
Apr-Jun 2020	247 (53.3%)	913 (13.8%)	0.79 †	229 (51.5%)	234 (52.6%)	0.02
Jul-Sep 2020	93 (20.1%)	642 (9.7%)	0.26 †	93 (20.9%)	97 (21.8%)	0.02
Oct-Dec 2020	29 (6.3%)	452 (6.9%)	0.02	29 (6.5%)	22 (4.9%)	0.06
Jan-Mar 2021	31 (6.7%)	1091 (16.5%)	0.39 †	31 (7.0%)	35 (7.9%)	0.04
Apr-Jun 2021	27 (5.8%)	1307 (19.8%)	0.60 †	27 (6.1%)	26 (5.8%)	0.01
Jul-Sep 2021	7 (1.5%)	455 (6.9%)	0.44 †	7 (1.6%)	5 (1.1%)	0.04
Oct-Dec 2021	3 (0.6%)	156 (2.4%)	0.21 †	3 (0.7%)	3 (0.7%)	0.00
Jan-Mar 2022	15 (3.2%)	916 (13.9%)	0.60 †	15 (3.4%)	11 (2.5%)	0.05
Apr-Jun 2022	1 (0.2%)	233 (3.5%)	0.71 †	1 (0.2%)	1 (0.2%)	0.00
Jul-Sep 2022	1 (0.2%)	108 (1.6%)	0.31 †	1 (0.2%)	3 (0.7%)	0.10
Oct-Dec 2022	0 (0.0%)	173 (2.6%)	0.17 †	0 (0.0%)	0 (0.0%)	0.00
Jan-Mar 2023	1 (0.2%)	70 (1.1%)	0.18 †	1 (0.2%)	0 (0.0%)	0.05
Apr-Jun 2023	1 (0.2%)	46 (0.7%)	0.10 †	1 (0.2%)	1 (0.2%)	0.00
Jul-Sep 2023	0 (0.0%)	8 (0.1%)	0.04	0 (0.2%)	0 (0.0%)	0.00

† Absolute standardised mean difference > 0.10, indicating imbalance in baseline covariates

Abbreviations: IQR, interquartile range; SpO2, peripheral oxygen saturation

We performed a sensitivity analysis using patients admitted within 14 days of symptom onset. All statistical analyses were done in RStudio version 4.4.1.

## Results

**Fig. 2:**
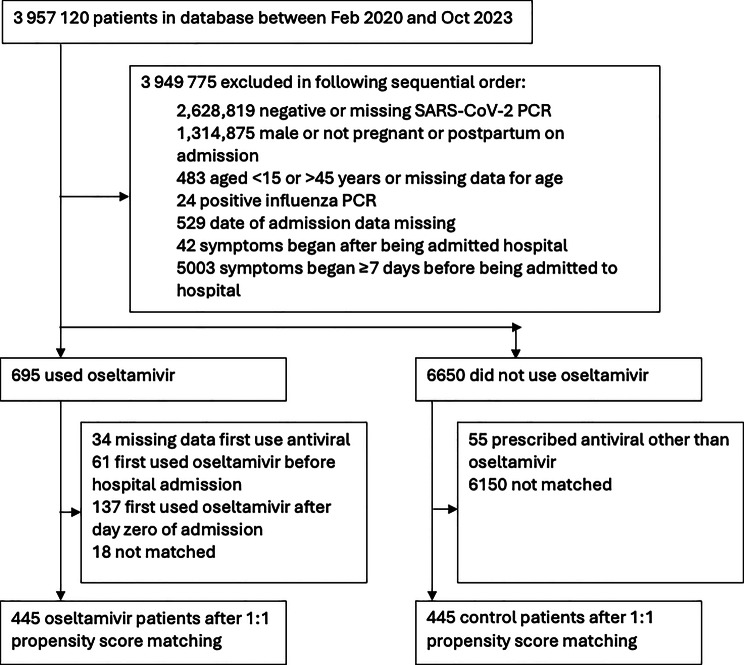
Patient selection procedure. *Abbreviations: PCR, polymerase chain reaction*

**Fig. 3:**
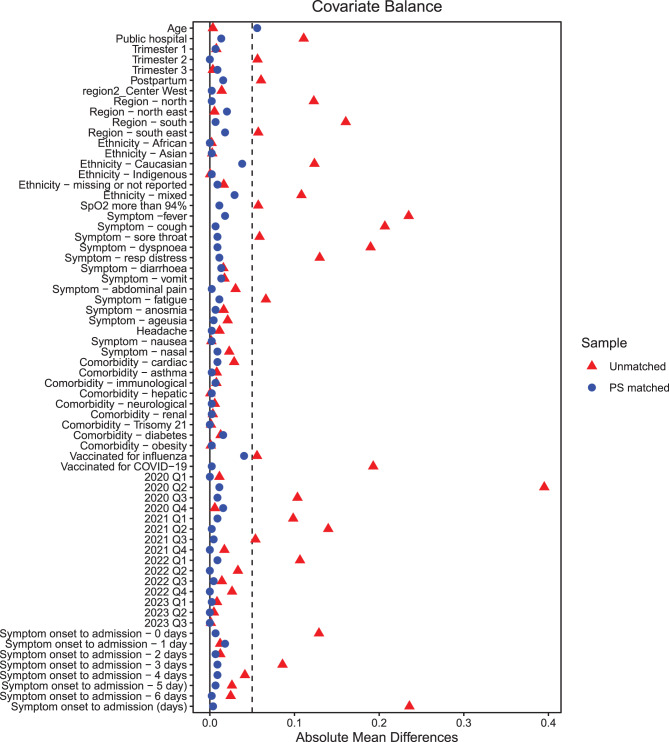
Love plot of the standardised mean differences of the unmatched and matched cohort

We identified 13,426 patients who were admitted to hospital between February 2020 and October 2023 and were PCR-positive for SARS-CoV-2 and were pregnant or postpartum and female (Fig. 2). Table 1 shows the baseline characteristics before 1:1 matching. After propensity score matching by baseline covariates with a calliper of 0.05, 445 oseltamivir recipients and 445 matched controls were included. Baseline characteristics were well balanced with SMD ≤ 0.10 (Fig. 3). Propensity score distributions of the two groups were highly overlapping (Supplementary F).

After matching, 79.5% oseltamivir recipients and 80.0% matched controls were admitted in 2020, 4.7% and 4.5% respectively were vaccinated for COVID-19, 48.1% and 47.2% were in third trimester and 65.8% and 67.9% had SpO2 > 94% on admission (Table 1). Median follow-up time was 7.0 days in oseltamivir recipients and 6.0 days in matched controls (Supplementary F). After matching, 5.8% oseltamivir recipients and 7.9% matched controls had missing clinical outcomes and were censored at 114 days (Supplementary F); and 1.6% and 1.8% participants respectively had missing discharge dates and their follow-up times were imputed.

**Fig. 4:**
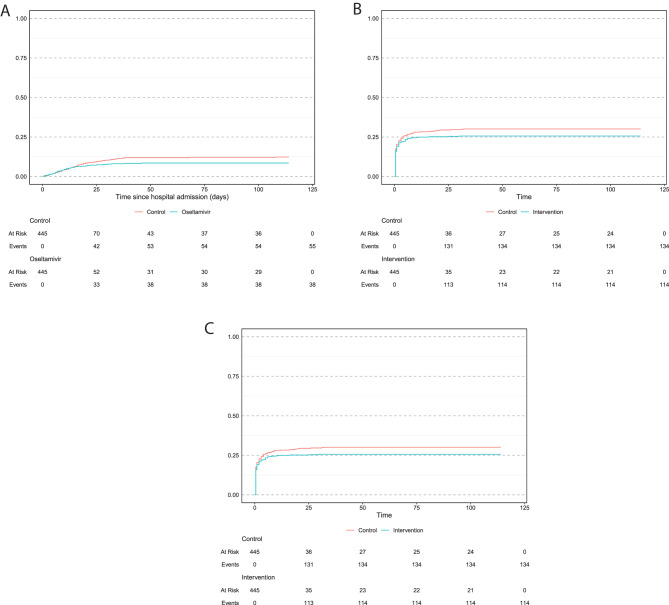
(**A**) Cumulative incidence functions of in-hospital death (all causes). (**B**) Cumulative incidence function of composite outcome (death or ICU admission). (**C**) Cumulative incidence function of hospital discharge for oseltamivir recipients and their matched controls.Death and discharge were competing risks & so we computed cause-specific hazard ratios. Those with missing data for the clinical endpoint were coded as censored. Censoring occurred at the end of the follow-up period

Oseltamivir use was associated with lower risk of dying in hospital (HR 0.77 [0.51–1.17], *p* = 0.22) than no antivirals, but the association was not statistically significant (Table 2). Figure 4 shows cumulative incidence functions. Cumulatively, 38 (8.5%) oseltamivir recipients and 55 (12.4%) matched controls died in-hospital, giving an ARR of 3.9% and NNT of 26 (Table 2).

**Table 2:** In-hospital death: hazard ratios over follow-up for oseltamivir recipients versus matched controls, overall and subgroups

In-hospital death	Oseltamivir (*n* = 445)	Controls (*n* = 445)	Oseltamivir versus controls			
	Events (%)	Events (%)	Cox: cause-specific HR (95% CI)	p	ARR	NNT
Overall	38/445 (8.5%)	55/445 (12.4%)	0.77 (0.51, 1.17)	0.22	3.9%	26
Admission date						
Before 1 Jan 2021	20/358 (5.6%)	39/360 (10.8%)	0.54 (0.32, 0.93)*	0.03*	5.2%	20
On or after 1 Jan 2021	18/87(20.7%)	16/85 (18.8%)	1.57 (0.77, 3.22)	0.21	−1.9%	n/a
SpO2 > 94% on admission						
Yes	5/293 (1.7%)	16/298 (5.4%)	0.33 (0.12, 0.89)*	0.03*	3.7%	28
No	33/152 (21.7%)	39/147 (26.5%)	1.04 (0.66, 1.65)	0.86	4.8%	21
Pregnant or postpartum						
Third trimester	13/214 (6.1%)	24/210 (11.4%)	0.52 (0.27, 1.03)	0.06	5.3%	19
Postpartum	19/94 (20.2%)	20/101 (19.8%)	1.41 (0.71, 2.60)	0.27	−0.4%	n/a
Vaccinated for COVID						
Yes	0/21 (0.0%)	0/20 (0.0%)	-	n/a	n/a	n/a
No	38/424 (9.0%)	55/425 (12.9%)	0.76 (0.51, 1.15)	0.19	3.9%	26

Abbreviations: ARR, absolute risk reduction; CI, confidence interval; HR, hazard ratio; n/a, not applicable; NNT, number needed to treat

*p ≤ 0.05

HR > 1 indicates hazard (i.e. instantaneous rate) of outcome was higher in oseltamivir group versus matched control group

Oseltamivir use was associated with a lower risk of progression to severe disease (HR 0.83 [0.67–1.01], *p* = 0.07) than no antivirals, but the association was not statistically significant. Cumulatively, 114 (25.6%) oseltamivir recipients and 134 (30.1%) matched controls progressed to severe disease (ICU admission or death), giving ARR 4.5% and NNT 23 (Table 3).

**Table 3:** Composite outcome: hazard ratios over follow-up for oseltamivir recipients versus matched controls, overall and subgroups

Composite outcome	Oseltamivir (*n* = 445)	Controls (*n* = 445)	Oseltamivir versus controls			
	Events (%)	Events (%)	Cox: cause-specific HR (95% CI)	p	ARR	NNT
Overall	114/445 (25.6%)	134/445 (30.1%)	0.83 (0.67, 1.01)	0.07	4.5%	23
Admission date						
Before 1 Jan 2021	84/358 (23.5%)	106/360 (29.4%)	0.76 (0.60, 0.97)*	0.03*	5.9%	17
On or after 1 Jan 2021	30/87 (34.5%)	28/85 (32.9%)	1.09 (0.69, 1.71)	0.71	−1.6%	n/a
SpO2 > 94% on admission						
Yes	37/293 (12.6%)	44/298 (14.8%)	0.83 (0.55, 1.25)	0.38	2.2%	46
No	77/152 (50.7%)	90/147 (61.2%)	0.80 (0.60, 1.05)	0.11	10.5%	10
Pregnant or postpartum						
Third trimester	44/214 (20.6%)	54/210 (25.7%)	0.76 (0.52, 1.10)	0.15	5.1%	20
Postpartum	43/94 (45.7%)	44/101 (43.6%)	1.09 (0.74, 1.63)	0.66	−2.1%	n/a
Vaccinated for COVID						
Yes	6/21 (28.6%)	4/20 (20.0%)	1.41 (0.40, 4.92)	0.59	−8.6%	n/a
No	108/424 (25.5%)	130/425 (30.6%)	0.80 (0.65, 0.99)*	0.04*	5.1%	20

Composite outcome: death or ICU admission, whichever occurred firstly

Abbreviations: ARR, absolute risk reduction; CI, confidence interval; HR, hazard ratio; ICU, intensive care unit; n/a, not applicable; NNT, number needed to treat

*p ≤ 0.05

HR > 1 indicates hazard (i.e. instantaneous rate) of outcome was higher in oseltamivir group versus matched control group

In the Cox model for time-to-hospital discharge, the proportional hazards assumption was not satisfied. We split the follow-up time into days 0–2 and days ≥3 from the index date based on the Schoenfeld residuals (Supplementary F). Oseltamivir recipients were less likely than controls to be discharged on days 0–2 (HR 0.68 [0.52–0.90]) but more likely to be discharged from days 3 onwards (HR 1.30 [CI 1.10–1.54]) (Table 4). Repeating analyses using the Fine-Gray model produced similar results for the primary and secondary outcomes (Supplementary F).

**Table 4:** Hospital discharge: hazard ratios for over follow-up for oseltamivir recipients versus matched controls, overall and subgroups

Hospital discharge	Oseltamivir (*n* = 445)	Control (*n* = 445)	Oseltamivir versus control	
	Events (%)	Events (%)	Cox: cause-specific HR (95% CI)	
			Days 0–2	Days ≥3
Overall	381/445 (85.6%)	355/445 (79.8%)	0.68 (0.51, 0.90)*	1.30 (1.09, 1.55)*
Admission date				
Before 1 Jan 2021	315/358 (88.0%)	295/360 (81.9%)	0.70 (0.52, 0.96)*	1.25 (1.03, 1.51)*
On or after 1 Jan 2021	66/87 (75.9%)	60/85 (70.6%)	1.17 (0.81, 1.69)¶	
SpO2 > 94% on admission				
Yes	267/293 (91.1%)	259/298 (86.9%)	0.69 (0.51, 0.94)*	1.18 (0.95, 1.46)
No	114/152 (75.0%)	96/147 (65.3%)	1.36 (1.05, 1.76)¶	
Pregnant or postpartum				
Third trimester	186/214 (86.9%)	174/210 (82.9%)	0.98 (0.80, 1.21)¶	
Postpartum	69/94 (73.4%)	65/101 (64.4%)	0.57 (0.31, 1.06)	1.83 (1.20, 2.81)*
Vaccinated for COVID				
Yes	20/21 (95.2%)	17/20 (85.0%)	1.27 (0.65, 2.45)¶	
No	361/424 (85.1%)	338/425 (79.5%)	0.68 (0.51, 0.91)*	1.28 (1.07, 1.52)*

Follow-up times are split

Abbreviations: CI, confidence interval; HR, hazard ratio

*p ≤ 0.05

¶ Proportional hazards assumption satisfied and so hazard ratio applies to entire follow-up period

HR > 1 indicates hazard (i.e. instantaneous rate) of outcome was higher in oseltamivir group versus matched control group

In the subgroup of patients admitted in 2020 (*n* = 718), oseltamivir use was associated with lower risk of dying in hospital (HR 0.54 [CI 0.32–0.93], *p* = 0.03) and of progression to severe disease (HR 0.76 [0.60–0.97], *p* = 0.03) and higher risk of being discharged from day 3 onwards (HR 1.25 [1.03–1.51]) than no antivirals (Tables 2–4). In the subgroup of patients with non-severe COVID-19 on admission (SpO2 > 94%) (*n* = 591), oseltamivir use was associated with lower risk of dying in hospital (HR 0.33 [0.12–0.89], *p* = 0.03), and with a non-significantly lower risk of progression to severe disease (HR 0.83 [0.55–1.25], *p* = 0.38). In the subgroup of patients admitted after 1 January 2021 (*n* = 172) or postpartum (*n* = 195), oseltamivir use was associated with higher risk of in-hospital death (HR 1.57 [0.77–3.22], *p* = 0.21; HR 1.41 [0.71–2.60], *p* = 0.71, respectively).

In sensitivity analyses of patients admitted within 14 days of symptom onset (*n* = 1266), oseltamivir use was associated with no differences in clinical outcomes compared with no antivirals in main analyses (Supplementary G). However, in the subgroup of patients admitted with non-severe COVID-19 (SpO2 > 94%) (*n* = 806), oseltamivir use was associated with lower risk of in-hospital death (HR 0.36 [0.17–0.77], *p* < 0.01) and progression to severe disease (HR 0.72 [0.52–0.99], *p* < 0.05).

On day 7 of follow-up (Supplementary F), 3.4% oseltamivir recipients versus 2.9% matched controls had died in hospital; 12.4% versus 14.8% respectively were admitted in ICU and 52.4% versus 53.0% were discharged. By day 28, 7.6% versus 10.1% had died in hospital; 2.7% versus 7.6% were in ICU and 82.0% versus 75.3% were discharged.

## Discussion

### Main findings

This was the first study focusing on obstetric patients to investigate the comparative effectiveness of oseltamivir (an anti-influenza medication) repurposed to treat COVID-19. Oseltamivir used within seven days of symptom onset was associated with a non-statistically significant lower risk of in-hospital death and progression to severe disease, compared with no antivirals. Oseltamivir recipients were significantly less likely to be discharged on days 0–2 but more likely to be discharged from days ≥3, compared with matched controls. While oseltamivir may have a role to complement SARS-CoV-2 treatment in obstetric patients, this study does not provide conclusive evidence to support its use.

### Interpretation

It is possible that the overall non-significant results for the study were due to subgroups in opposite directions, with the HRs in those admitted in 2020 versus 2021 in opposite directions. In the subgroup of patients admitted in 2020 (*n* = 718/890), oseltamivir use was associated with statistically significantly lower risk of in-hospital death and progression to severe disease.

There is not enough evidence to support the positive association of oseltamivir use with mortality from 2021 onwards (*n* = 172). The sample sizes were too small and the confidence intervals were wide, with only two more mortality events in the oseltamivir group than the control group (Table 2). Also the Fine Gray model showed no difference in cumulative incidence of deaths (Supplementary F).

In main analyses, oseltamivir patients were less likely to be discharged at 0–2 days and more likely to be discharged ≥3 days. Clinical benefits may have presented after a few days of treatment. A recently published study for oseltamivir in a non-obstetric patients with COVID-19 [12] showed the majority of clinical indices were better on day 4 in the intervention group, even if no differences existed in indices at the index date. Alternatively, it is possible that oseltamivir patients were slightly more ill at baseline compared with matched controls, which could have delayed their discharge. However in the SIVEP-Gripe, severities in laboratory data were not measured. The hospital discharge findings, however, do not support routine use of oseltamivir.

In sensitivity analyses, the lack of overall effectiveness of oseltamivir when used up to 14 days after symptom onset was likely due to the dilution of the overall effect by mixing patients admitted from 7 to 14 days with those admitted 0–7 days after symptom onset. In comparison, Paxlovid® and remdesivir are associated with improved clinical outcomes when given within 5–7 days of symptom onset of COVID-19[20].

Other studies investigated comparative effectiveness of oseltamivir in general (non-obstetric) populations. One study was a retrospective cohort study that investigated outcomes of patients admitted to a Chinese hospital from January to April 2020 (*n* = 972 in the matched cohort) [12]. Initiation of neuraminidase inhibitors (oseltamivir, zanamivir, peramivir or other) was associated with a lower risk of in-hospital all-cause mortality (HR 0.36 [0.19–0.68]) compared with no antivirals [12]. Another retrospective cohort study used data from the SIVEP-Gripe of hospitalised patients in Brazil (*n* = 289,966 in the matched cohort) [11]. Oseltamivir use was associated with lower odds of in-hospital mortality compared with matched controls (odds ratio 0.90 [0.87–0.93]) [11]. Findings from other studies [33] are difficult to interpret because of small sample sizes and/or lack of suitable control groups.

In terms of possible mechanisms, SARS-CoV-2 does not contain neuraminidase and so oseltamivir, which is a neuraminidase inhibitor, was initially not thought to be effective. One study showed that the neuraminidase-1 enzyme in human cells may be important in the SARS-CoV-2 entry into host cells, and so oseltamivir may instead act as a host-targeting agent when used to treat COVID-19 [34]. Another study showed that neuraminidase inhibitors may dampen neutrophil dysfunction in severe COVID-19 [35].

Findings in certain subgroups are noteworthy and warrant cautious interpretation. Oseltamivir was associated with lower mortality risk compared with no antivirals in subgroup of those SpO2 > 94% at baseline in main and sensitivity analyses, which was an important finding. Low oxygen saturations (hypoxia) are a sign of respiratory decompensation and indicate that the patient may have passed from the acute viral phase into the hyperinflammatory phase of the illness [36]. Paxlovid® and remdesivir are most effective when given in those without hypoxia [1, 37].

It is possible that the positive associations of oseltamivir use with mortality from 2021 onwards (*n* = 172/890) were due to residual confounding by indication. This is despite that we matched on a wide range of baseline covariates, with excellent balance in baseline covariates. There was a shift away from prescribing oseltamivir from 2021 onwards in Brazil, with changing standards of care, the availability of COVID-19 specific vaccinations and treatments and policymakers citing the lack of efficacy of oseltamivir [5, 6]. Supplementary Table F7 shows the baseline characteristics of oseltamivir recipients and matched controls in the subgroup admitted in 2020 versus 2021 onwards.

Selection bias may also have contributed. The Gamma variant of SARS-CoV-2 became prevalent in 2021, which was more virulent. Pregnant and postpartum patients in Brazil with COVID-19 admitted in 2021, compared with in 2020, had higher morbidity and mortality rates [38, 39], which reflected experiences in other countries [40]. This was despite increased healthcare knowledge of the disease and initiation of COVID-19 vaccinations from 2021. With increased healthcare pressure, there was likely a reduced ability to provide adequate care for patients and an increase in the average severity of admitted patients and, during periods of highest pressure, with only the most severely ill being admitted[41].

### Strength and limitations

Although primary and secondary outcomes did not reach statistical significance and the confidence intervals were wide, this paper is methodologically robust and we believe contributes valuable evidence regarding oseltamivir use in obstetric patients with COVID-19. In terms of study strengths, we defined the index date as the date of hospitalisation, which coincided with the date of treatment exposure. This prevented immortal time bias [21]. We used a national database with high completion rates and so our cohort was highly representative of the Brazilian hospitalised obstetric population. Patients received oseltamivir within 5–7 days of symptom onset, which reflected prescribing guidelines for Paxlovid® and remdesivir [20]. Oseltamivir is used widely in pregnancy and so the study was unlikely to be influenced by patient preference to avoid oseltamivir.

Study limitations included the small sample size of the matched cohort (*n* = 890). This limited power for statistical analyses, especially for mortality which was a rare outcome. There may have been residual confounding in the subgroup of patients admitted from 2021, as discussed above. The significant results pertained to the first year of the pandemic with data from one country, Brazil, in an unvaccinated population. This may limit generalisability to other settings. We excluded patients who were initiated oseltamivir as outpatients (*n* = 66), because we needed to align treatment initiation with the start of follow-up to focus on new users of oseltamivir and to create a common baseline for follow-up. People from the North and Northeast regions of Brazil were likely under-represented in this cohort. Socioeconomic problems in those regions probably reduced the rate of PCR-testing for COVID-19, number of hospitalisations and access to therapeutic resources [31]. The database coded for anti-influenza antivirals (oseltamivir, zanamivir and ‘other’) and did not code for other medications, which limited comparisons with COVID-19 specific treatments. This however was unlikely to affect results as Paxlovid was available in Brazil from 2022 only.

Causal interpretations of our study conclusions rely on the standard assumptions of propensity score analyses, including no unmeasured confounding, positivity and consistency. Although we included a comprehensive set of covariates, the possibility of residual unmeasured confounding remains.

## Conclusions

In this retrospective cohort study, initiation of oseltamivir (an anti-influenza antiviral) within seven days of symptom onset in obstetric patients with COVID-19 were associated with non-statistically significant lower risks of all-cause mortality and progression to severe disease compared with no antivirals. Significant results were observed only in subgroups, a Brazilian population admitted in 2020 or those with SpO2 > 94% at treatment initiation. Oseltamivir recipients were more likely than matched controls to remain hospitalised during days 0–2 and to be discharged alive from days ≥3. Overall, results do not support the use of oseltamivir as a low-cost antiviral to treat COVID-19 in obstetric patients.

## Electronic supplementary material

Below is the link to the electronic supplementary material.

Supplementary Material 1

## Data Availability

The dataset supporting the conclusions of this article is available in the OSF Home repository: https://osf.io/uykdm/?view_only=308dd2ce1c5745a18ee55a91f247fe21

## Abbreviations

ARR: Absolute risk reduction
CI: Confidence interval
COVID-19: Coronavirus disease
HR: Hazard ratios
ICU: Intensive care unit
NNT: Number needed to treat
RCT: Randomised controlled trial
SIVEP-Gripe: Sistema de Informação de Vigilância Epidemiológica da Gripe (Influenza Epidemiological Surveillance Information System)
SMD: Standardised mean differences
WHO: World Health Organisation
