# Supplementary material for: Effectiveness of oseltamivir in hospitalised obstetric patients with COVID-19: a retrospective cohort study using a Brazilian national database
Source: BMC Infect Dis. 2025 Dec 20;26:135. doi: 10.1186/s12879-025-12327-x (PMC12831268; doi:10.1186/s12879-025-12327-x)
Supplement: Supplementary file 1 — Supplementary Material 1 [file 12879_2025_12327_MOESM1_ESM.docx]

**Effectiveness of oseltamivir in hospitalised obstetric patients with COVID-19: a retrospective cohort study using a Brazilian national database - supplementary material**

**Table of contents**

[Supplementary A](#appendix_A) STROBE guidelines for reporting cohort studies

[Supplementary B](#appendix_B) Brazilian Ministry of Health guidelines 2020 and 2021

[Supplementary C](#appendix_new_C) Further details on methods

[Supplementary D](#appendix_D) Baseline covariates from the SIVEP-Gripe

[Supplementary E](#appendix_new_E) Clinical states and possible transitions between states

[Supplementary F](#appendix_new_F) Main analysis – supplementary figures and tables

[Supplementary G](#supplementary_G) Sensitivity analysis – admitted <14 days symptom onset

[Supplementary H](#supplementary_H) Dataset & R code

[References](#References)

**Supplementary A**. STROBE guidelines for reporting cohort studies (1)

|  | Recommendation | Section |
| --- | --- | --- |
| Title and abstract |  |  |
|  | (1a) Indicate the study’s design with a commonly used term in the title or the abstract | Title |
|  | (1b) Provide in the abstract an informative and balanced summary of what was done and what was found | Abstract |
| Introduction |  |  |
| Background  /rationale | (2) Explain the scientific background and rationale for the investigation being reported | 1 |
| Objectives | (3) State specific objectives, including any prespecified hypotheses | 1 |
| Methods |  |  |
| Study design | (4) Present key elements of study design early in the paper | 2.1 |
| Setting | (5) Describe the setting, locations, and relevant dates, including periods of recruitment, exposure, follow-up, and data collection | 2.2, 2.3 |
| Participants | (6a) Give the eligibility criteria, and the sources and methods of selection of participants. Describe methods of follow-up | 2.2, 2.3 |
|  | (6b) For matched studies, give matching criteria and number of exposed and unexposed | 2.6 |
| Variables | (7) Clearly define all outcomes, exposures, predictors, potential confounders, and effect modifiers. Give diagnostic criteria, if applicable | 2.3, 2.4, 2.5 |
| Data sources/ measurement | (8) For each variable of interest, give sources of data and details of methods of assessment (measurement). Describe comparability of assessment methods if there is more than one group | 2.2, 2.3, 2.4, 2.5 |
| Bias | (9) Describe any efforts to address potential sources of bias | 2.6 |
| Study size | (10) Explain how the study size was arrived at | 2.2 |
| Quantitative variables | (11) Explain how quantitative variables were handled in the analyses. If applicable, describe which groupings were chosen and why | 2.5 |
| Statistical methods | (12a) Describe all statistical methods, including those used to control for confounding | 2.6 |
|  | (12b) Describe any methods used to examine subgroups and interactions | 2.6 |
|  | (12c) Explain how missing data were addressed | 2.2, 2.3, 2.5 |
|  | (12d) If applicable, explain how loss to follow-up was addressed | 2.3 |
|  | (12e) Describe any sensitivity analyses | 2.6 |
| Results |  |  |
| Participants | (13a) Report numbers of individuals at each stage of study—e.g. numbers potentially eligible, examined for eligibility, confirmed eligible, included in the study, completing follow-up, and analysed | Figure 2 |
|  | (13b) Give reasons for non-participation at each stage | Figure 2 |
|  | (13c) Consider use of a flow diagram | Figure 2 |
| Descriptive data | (14a) Give characteristics of study participants (e.g. demographic, clinical, social) and information on exposures and potential confounders | Table 1 |
|  | (14b) Indicate number of participants with missing data for each variable of interest | Table 1  Supplementary |
|  | (14c) Summarise follow-up time (e.g., average and total amount) | Supplementary |
| Outcome data | (15) Report numbers of outcome events or summary measures over time | Tables 2, 3, 4 |
| Main results | (16a) Give unadjusted estimates and, if applicable, confounder-adjusted estimates and their precision (e.g., 95% confidence interval). Make clear which confounders were adjusted for and why they were included | Tables 2, 3, 4 |
|  | (16b) Report category boundaries when continuous variables were categorized | Table 1 |
|  | (16c) If relevant, consider translating estimates of relative risk into absolute risk for a meaningful time period | Tables 2, 3 and 4 |
| Other analyses | (17) Report other analyses done—e.g. analyses of subgroups and interactions, and sensitivity analyses | Tables 2, 3 and 4  Supplementary |
| Discussion |  |  |
| Key results | (18) Summarise key results with reference to study objectives | 4.1 |
| Limitations | (19) Discuss limitations of the study, taking into account sources of potential bias or imprecision. Discuss both direction and magnitude of any potential bias | 4.3 |
| Interpretation | (20) Give a cautious overall interpretation of results considering objectives, limitations, multiplicity of analyses, results from similar studies, and other relevant evidence | 4.2, 4.4 |
| Generalisability | (21) Discuss the generalisability (external validity) of the study results | 4.3 |
| Other information |  |  |
| Funding | (22) Give the source of funding and the role of the funders for the present study and, if applicable, for the original study on which the present article is based | Acknowledgements |

**Supplementary B**. Recommendations for medications to prescribe in pregnant women with COVID-19 according to severity of disease, translated and adapted from 2020 and 2021 Brazilian Ministry of Health guidelines (2, 3)

| Stage | 2020 guidelines | 2021 guidelines |
| --- | --- | --- |
| Asymptomatic | No medications | No medications |
| Mild | Outpatient medications   - Oseltamivir: if flu-like syndrome with onset less than 48 hours ago - Supportive measures (rest, hydration, analgesics, antipyretics) | Outpatient medications   - Oseltamivir**:** if flu-like syndrome with onset less than 48 hours ago - Supportive measures (rest, hydration, analgesics, antipyretics) |
| Moderate | Hospital medications   - Antibiotics: if a sign of bacterial infection - Oseltamivir: if flu-like syndrome started less than 48 hours ago - Heparin - Corticosteroid therapy | Hospital medications   - Antibiotics: if a sign of bacterial infection - Oseltamivir**:** if flu-like syndrome started less than 48 hours ago - Heparin - Corticosteroid therapy |
| Severe | ICU medications   - Antibiotics - Oseltamivir - Heparin - Corticosteroid therapy | ICU medications   - Antibiotics - Heparin - Corticosteroid therapy |

**Supplementary C**. Further details on methods

**2.3 Treatment exposure and follow-up**

***Those with missing data for clinical endpoint***

Those with missing data for the clinical endpoint were coded as censored. These patients were assumed to remain in hospital until the censoring time. We defined the censoring time as the maximum length of hospital stay in the matched cohort (114 days) or until October 2023 (the date of data extraction), whichever was earlier.

***Those with missing data for date of discharge***

The SIVEP-Gripe database recorded some patients as ‘discharged’ but they had missing discharge dates. For those with missing data for date of discharge (n=15), the length of hospital stay was imputed based on the median values of time to discharge of those who were in the same calendar time period (2020 or 2021 onwards), treatment group and time from symptom onset category (<7 days or ≥7 days), as these were the main baseline factors known to likely affect time to hospital discharge.

There were six participants in the matched cohort whose date of discharge or death was recorded over one year from their ‘date of admission’. Errors in data entry are common in national registries. The errors likely occurred in the year recorded, which we corrected. For example, if the year of admission was 2020 and the year of clinical endpoint was 2022, it was changed to 2020, since it was unlikely that a patient was discharged or died greater than a year after diagnosis.

**2.5 Baseline covariates**

***Choice of baseline covariates – further information***

A wide range of covariates that may be associated with treatment assignment and/or the outcomes should be included in propensity score matching. (4) There is little cost to including variables that are not associated with treatment assignment because these will be of little influence in the propensity score model. (4) We therefore matched on a wide range of covariates. We matched only on baseline covariates, that is, those measured before treatment assignment.

**2.6 Statistical analysis**

***Further details on propensity score matching and methods***

To estimate causal effects using observational data, it is best to replicate a randomised experiment.(5) Randomised assignment mechanisms ensure balance in baseline covariates between the intervention and controlled groups.(4) Propensity score matching is a method to mimic a randomised controlled trial(4) The propensity score is the probability of the treatment being assigned, condition on observed (pre-exposure) baseline covariates.

Propensity score matching forms a set of treated and untreated patients who share a similar value of the propensity score. This aims to address confounding biases by balancing observed baseline covariates between groups. If the observed patient characteristics between groups were well balanced at baseline, under the unconfoundedness assumption and after using propensity score matching, one can estimate the treatment effect in the matched sample by directly comparing outcomes between the two groups. (5)

***Choice of matching ratio and calliper width***

The matching ratio of 1:1 was chosen, which is the standard ratio for randomisation in clinical trials. (4)

Adding a calliper width to the matching method meant that, for every patient in the treatment group, patients in the control group can only be chosen if their propensity scores are within a limited range from the treated patient’s propensity score. If a treated patient cannot be matched, they are discarded. There is no gold standard for the maximal acceptable difference for determining the calliper. (6) Calliper width 0.05 was chosen because this is the value used in other studies with similar settings. (7-9)

***Propensity score matching – R code***

The R code for the propensity score matching is below:

m.out1 <- matchit(Tamiflu ~ public + quarter2 + time_admission_Sx*s.fever + time_admission_Sx*s.cough + time_admission_Sx*s.headache + age + time_admission_Sx2 + ethnicity3 + region2 + trimester2 + s.fever + s.cough + s.sorethroat + s.dyspnea + s.spo2 + s.resp.disc + s.diarrhea + s.vomit + s.abd.pain + s.fatigue + s.anosmia + s.ageusia + s.headache + s.nausea + s.nasal + c.cardio + c.asthma + c.immuno + c.liver + c.neuro + c.renal + c.down + c.diabe + c.obesity + vac.flu + vac.covid, data=data_main, method="nearest", distance="glm", replace=FALSE, ratio=1, caliper=0.05)

The terms ‘time_admission_Sx*s.fever’, ‘time_admission_Sx*s.cough’, ‘time_admission_Sx*s.headache’ were the interaction terms between *‘time from symptom onset’ and ‘fever’, ‘cough’ and ‘headache’.*

For the covariates with more than two categories, these were treated as categorical variables in the logistic regression analysis.

***Proportional hazards assumption***

The proportional hazards assumption is not satisfied where a covariate is measured at baseline but its effect on the outcome is not constant over the follow-up time. (10)

For each Cox regression model, we assessed the proportional hazards assumption by hypothesis testing. We used the cox.zph() function in the *survival* package in R, which gave the Schoenfeld’s global test for the violation of the proportional assumption. (10) We rejected the null hypothesis of proportional hazards with a significance level of 0.05. We plotted the Schoenfeld residuals, to visually assess the change in the hazard ratio of oseltamivir over time. (10)

Where the proportional hazards assumption was not satisfied, we accounted for the time-varying exposure effects. The follow-up time was split into intervals and the hazard ratios of the intervention was allowed to vary across these intervals, resulting in a series of time-varying hazard ratios. (10) We chose the cut-offs of the time intervals based on the Schoenfeld residual plots. Specifically, the cut-offs corresponded to the time where the time-varying hazard ratio estimated in the Schoenfeld residuals plots crossed zero and changed signs.

***Cumulative incidence functions***

Cumulative incidence functions were presented as non-parametric summary of outcome distributions. Competing risks were accounted for in the cumulative incidence curves. Figure 4A shows the cumulative incidence of ‘in-hospital death’. The competing risk ‘hospital discharge’ was reflected when calculating the risk set of ‘in-hospital death’ in this plot. Similarly, Figure 4C gives the cumulative incidence of ‘hospital discharge’, with ‘in-hospital death’ accounted for when calculating the corresponding risk set.

***Fine Gray sub-distribution HR***

For studies with competing risks and propensity score matching, treatment effect estimates using Cox models and Fine Gray models complement each other.(12) This is a method estimate the hazard ratio in survival analysis with competing risks in the clinical setting. This was to complement treatment effect estimates using Cox models.

***Software***

All statistical analyses were performed in RStudio version 4.4.1 using the following packages: survival, flexsurv, skimr, survminer, tidyverse, MatchIt, grDevices, cmprsk, riskRegression, prodlim, ggsurvfit, nortest and cobalt.

**Supplementary D**. Baseline covariates from SIVEP-Gripe dataset

| Baseline covariate | Values / categories | How groupings were chosen (if applicable) |
| --- | --- | --- |
| Sociodemographic factors | | |
| Age (at symptom onset) | Continuous |  |
| Type of hospital admitted | Public  Private | SIVEP Gripe gave four categories: public, private, privately managed public hospital, other (considered private).  We defined the latter three categories as ‘private’. |
| Brazilian region of residence (self-reported) | Centre West  North  Northeast  South  Southeast |  |
| Ethnicity (self-reported) | African  Asian  Caucasian  Indigenous  Mixed  Missing | There were missing data for this covariate. We created a new variable ‘missing or not reported’. |
| Pregnancy stage |  |  |
| Pregnant or postpartum (at admission) | First trimester  Second trimester  Third trimester  Postpartum (within 6 weeks of delivery) |  |
| Signs (reported by medical practitioner at admission) | | |
| Oxygen saturation SpO2 >94% | Yes / no |  |
| Symptoms (self-reported at admission) | | |
| Fever | Yes / no |  |
| Cough | Yes / no |  |
| Sore throat | Yes / no |  |
| Dyspnoea | Yes / no |  |
| Respiratory discomfort | Yes / no |  |
| Diarrhoea | Yes / no |  |
| Vomiting | Yes / no |  |
| Abdominal pain | Yes / no |  |
| Fatigue | Yes / no |  |
| Anosmia | Yes / no |  |
| Ageusia | Yes / no |  |
| Headache | Yes / no |  |
| Nausea | Yes / no |  |
| Nasal congestion | Yes / no |  |
| Comorbidities (reported by medical practitioner at admission) | | |
| Cardiovascular disease | Yes / no |  |
| Diabetes | Yes / no |  |
| Obesity | Yes / no |  |
| Asthma | Yes / no |  |
| Immunocompromise | Yes / no |  |
| Chronic liver disease | Yes / no |  |
| Chronic neurological disease | Yes / no |  |
| Chronic renal disease | Yes / no |  |
| Down syndrome | Yes / no |  |
| Vaccinations | | |
| Vaccinated against SARS-CoV-2 | Yes / no |  |
| Vaccinated against influenza | Yes / no |  |
| Dates | | |
| Time from symptom onset to admission | Days | We calculated the difference between ‘date of admission’ and ‘date of symptom onset’. |
| Calendar quarter of admission | January-March 2020  April-June 2020  July-September 2020  October-December 2020  January-March 2021  April-June 2021  July-September 2021  October-December 2021  January-March 2022  April-June 2022  July-September 2022  October-December 2022  January-March 2023  April-June 2023  July-September 2023 | We calculated the calendar quarter based on the ‘date of admission’. |

**Supplementary E.** Clinical states and possible transitions between states


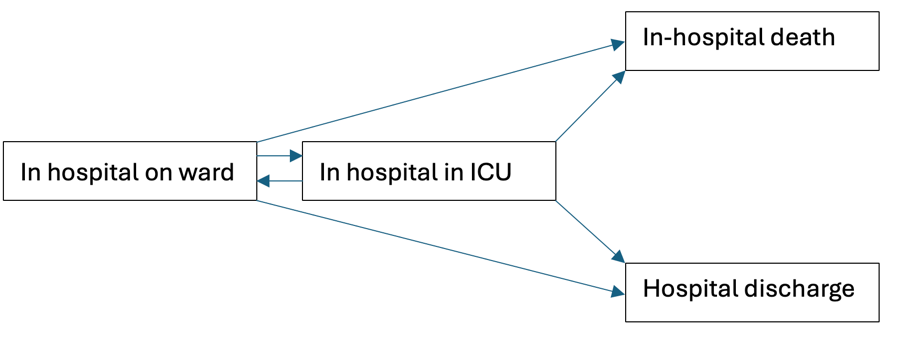


**Supplementary F.** Main analysis – supplementary figures and tables

**Figure F1**. Distribution of propensity scores after matching

**Table F1.** Follow-up times – overall and by subgroups

|  | **Oseltamivir recipients (n=445)** | | **Matched controls (n=445)** | |
| --- | --- | --- | --- | --- |
|  | **n** | **Follow-up**  **Median (mean)**  **(days)^** | **n** | **Follow-up**  **Median (mean)**  **(days)^** |
| Overall | 445 | 7.0 (15.7) | 445 | 6.0 (17.8) |
| Admission date |  |  |  |  |
| Before 1 Jan 2021 | 358 | 6.0 (16.3) | 360 | 6.0 (17.2) |
| On or after 1 Jan 2021 | 87 | 7.0 (13.6) | 85 | 8.0 (20.4) |
| SpO2 >94% on admission |  |  |  |  |
| Yes | 293 | 6.0 (15.5) | 298 | 5.0 (15.7) |
| No | 152 | 8.0 (16.2) | 147 | 9.0 (22.1) |
| Pregnant or postpartum |  |  |  |  |
| Third trimester | 214 | 7.0 (16.5) | 210 | 6.0 (15.3) |
| Postpartum | 94 | 8.0 (16.6) | 101 | 8.0 (27.0) |
| Vaccinated for COVID |  |  |  |  |
| Yes | 21 | 6.0 (11.6) | 20 | 5.5 (22.5) |
| No | 424 | 7.0 (16.0) | 425 | 6.0 (17.6) |

**Table F2**. Missing data for outcomes after matching

|  | **Oseltamivir (n= 445)** | **Control (n= 445)** |
| --- | --- | --- |
|  | **n (%)** | **n (%)** |
| Clinical outcome not available or missing* | 26 (5.8%) | 35 (7.9%) |
| Clinical outcome ‘discharged’ but missing date of discharge | 7 (1.6%) | 8 (1.8%) |

*Note: all of those who had missing data for missing outcome also did not have data for date of clinical endpoint

The 26 (5.8%) oseltamivir recipients and 35 (7.9%) controls who had missing data for clinical outcomes were coded as censored at 114 days, which was the maximum in-hospital length of stay in that cohort.

**Figure F2**. Schoenfeld residuals for in-hospital death (all-causes) for oseltamivir recipients and their matched controls

**Figure F3.** Schoenfeld residuals for composite outcome (death or ICU admission, whichever occurred firstly) for oseltamivir recipients and their matched controls

**Figure F4.** Schoenfeld residuals for hospital discharge (alive) for oseltamivir recipients and their matched controls

**Table F3**. Hazard ratios for **in-hospital death** (all-causes) over entire follow-up for oseltamivir recipients versus their matched controls, overall and by subgroups; with Cox cause-specific HRs and Fine-Gray sub distributional HRs

|  | **Oseltamivir recipients (n=445)** | **Matched controls (n=445)** | **Oseltamivir recipients versus matched controls** | | | | | |
| --- | --- | --- | --- | --- | --- | --- | --- | --- |
| **In-hospital death** | **Events (%)** | **Events (%)** | **Cox: cause-specific HR**  **(95% CI)** | **p** | **Fine-Gray: sub-distribution HR (95% CI)** | **p** | **ARR** | **NNT** |
| Overall | 38 / 445 (8.5%) | 55 / 445 (12.4%) | 0.77 (0.51, 1.17) | 0.22 | 0.68 (0.45, 1.04) | 0.07 | 3.9% | 26 |
| Admission date |  |  |  |  |  |  |  |  |
| Before 1 Jan 21 | 20 / 358 (5.6%) | 39 / 360 (10.8%) | 0.54 (0.32, 0.93) | 0.03† | 0.51 (0.29, 0.87) | 0.01† | 5.2% | 20 |
| On or after 1 Jan 21 | 18 / 87(20.7%) | 16 / 85 (18.8%) | 1.57 (0.77, 3.22) | 0.21 | 1.14 (0.57, 2.30) | 0.70 | -1.9% | n/a |
| SpO2 >94% |  |  |  |  |  |  |  |  |
| Yes | 5 / 293 (1.7%) | 16 / 298 (5.4%) | 0.33 (0.12, 0.89) | 0.03† | 0.31 (0.11, 0.86) | 0.02† | 3.7% | 28 |
| No | 33 / 152 (21.7%) | 39 / 147 (26.5%) | 1.04 (0.66, 1.65) | 0.86 | 0.82 (0.52, 1.30) | 0.40 | 4.8% | 21 |
| Stage pregnancy |  |  |  |  |  |  |  |  |
| Third trimester | 13 / 214 (6.1%) | 24 / 210 (11.4%) | 0.52 (0.27, 1.03) | 0.06 | 0.51 (0.26, 1.02) | 0.06 | 5.3% | 19 |
| Postpartum | 19 / 94 (20.2%) | 20 / 101 (19.8%) | 1.41 (0.71, 2.60) | 0.27 | 1.08 (0.59, 1.98) | 0.80 | -0.4% | n/a |
| COVID vaccinated |  |  |  |  |  |  |  |  |
| Yes | 0 / 21 (0.0%) | 0 / 20 (0.0%) | - | n/a | - | - | n/a | n/a |
| No | 38 / 424 (9.0%) | 55 / 425 (12.9%) | 0.76 (0.51, 1.15) | 0.19 | 0.69 (0.45, 1.04) | 0.08 | 3.9% | 26 |

*Abbreviations: ARR, absolute risk reduction; CI, confidence interval; HR, hazard ratio; n/a, not applicable; NNT, number needed to treat.*

*†: p ≤ 0.05.*

*HR >1 indicates hazard (i.e. instantaneous rate) of outcome was higher in oseltamivir group versus matched control group.*

**Table F4**. Hazard ratios for **composite outcome** (death or ICU admission, whichever occurred firstly) over entire follow-up for oseltamivir recipients versus their matched controls, overall and by subgroups; with Cox cause-specific HRs and Fine-Gray sub distributional HRs

|  | **Oseltamivir recipients (n=445)** | **Matched controls (n=445)** | **Oseltamivir recipients versus matched controls** | | | | | |
| --- | --- | --- | --- | --- | --- | --- | --- | --- |
| **Composite outcome** | **Events (%)** | **Events (%)** | **ARR** | **NNT** | **Cox: cause-specific HR**  **(95% CI)** | **p** | **Fine-Gray: sub-distribution HR (95% CI)** | **p** |
| Overall | 114 / 445 (25.6%) | 134 / 445 (30.1%) | 4.5% | 23 | 0.83 (0.67, 1.01) | 0.07 | 0.84 (0.69, 1.02) | 0.08 |
| Admission date |  |  |  |  |  |  |  |  |
| Before 1 Jan 21 | 84 / 358 (23.5%) | 106 / 360 (29.4%) | 5.9% | 17 | 0.76 (0.60, 0.97) | 0.03† | 0.78 (0.62, 0.98) | 0.03† |
| On or after 1 Jan 21 | 30 / 87 (34.5%) | 28 / 85 (32.9%) | -1.6% | n/a | 1.09 (0.69, 1.71) | 0.71 | 1.07 (0.70, 1.65) | 0.75 |
| SpO2 >94% |  |  |  |  |  |  |  |  |
| Yes | 37 / 293 (12.6%) | 44 / 298 (14.8%) | 2.2% | 46 | 0.83 (0.55, 1.25) | 0.38 | 0.85 (0.57, 1.27) | 0.43 |
| No | 77 / 152 (50.7%) | 90 / 147 (61.2%) | 10.5% | 10 | 0.80 (0.60, 1.05) | 0.11 | 0.79 (0.62, 1.02) | 0.07 |
| Stage pregnancy |  |  |  |  |  |  |  |  |
| Third trimester | 44 / 214 (20.6%) | 54 / 210 (25.7%) | 5.1% | 20 | 0.76 (0.52, 1.10) | 0.15 | 0.79 (0.55, 1.13) | 0.19 |
| Postpartum | 43 / 94 (45.7%) | 44 / 101 (43.6%) | -2.1% | n/a | 1.09 (0.74, 1.63) | 0.66 | 1.08 (0.76, 1.54) | 0.66 |
| COVID vaccinated |  |  |  |  |  |  |  |  |
| Yes | 6 / 21 (28.6%) | 4 / 20 (20.0%) | -8.6% | n/a | 1.41 (0.40, 4.92) | 0.59 | 1.46 (0.45, 4.76) | 0.53 |
| No | 108 / 424 (25.5%) | 130 / 425 (30.6%) | 5.1% | 20 | 0.80 (0.65, 0.99) | 0.04† | 0.82 (0.67, 1.00) | 0.05† |

*Abbreviations: ARR, absolute risk reduction; CI, confidence interval; HR, hazard ratio; ICU, intensive care unit; n/a, not applicable; NNT, number needed to treat.*

*†: p ≤ 0.05.*

*HR >1 indicates hazard (i.e. instantaneous rate) of outcome was higher in oseltamivir group versus matched control group.*

**Table F5**. Hazard ratios for **time-to-hospital discharge** (alive) over entire follow-up for oseltamivir recipients versus their matched controls, overall and by subgroups, with split follow-up times; with Cox cause-specific HRs and Fine-Gray sub distributional HRs

|  | **Oseltamivir recipients (n=445)** | **Matched controls (n=445)** | **Oseltamivir recipients versus matched controls** | | | |
| --- | --- | --- | --- | --- | --- | --- |
| Hospital discharge (alive) | Events (%) | Events (%) | Cox: cause-specific HR (95% CI) | | Fine-Gray: sub-distribution HR (95% CI) | |
|  |  |  | Days 0-2 | Days ≥3 | Days 0-2 | Days ≥3 |
| Overall | 381 / 445 (85.6%) | 355 / 445 (79.8%) | 0.68 (0.51, 0.90)† | 1.30 (1.09, 1.55)† | 0.54 (0.37, 0.77)† | 1.21 (1.03, 1.42)† |
| Admission date |  |  |  |  |  |  |
| Before 1 Jan 21 | 315 / 358 (88.0%) | 295 / 360 (81.9%) | 0.70 (0.52, 0.96)† | 1.25 (1.03, 1.51)† | 0.55 (0.37, 0.81)† | 1.21 (1.02, 1.45)† |
| On or after 1 Jan 21 | 66 / 87 (75.9%) | 60 / 85 (70.6%) | 1.17 (0.81, 1.69)¶ |  | 1.08 (0.76, 1.54)¶ |  |
| SpO2 >94% |  |  |  |  |  |  |
| Yes | 267 / 293 (91.1%) | 259 / 298 (86.9%) | 0.69 (0.51, 0.94)† | 1.18 (0.95, 1.46) | 0.61 (0.41, 0.91)† | 1.13 (0.94, 1.37)† |
| No | 114 / 152 (75.0%) | 96 / 147 (65.3%) | 1.36 (1.05, 1.76)¶† |  | 1.25 (0.96, 1.62)¶ |  |
| Stage pregnancy |  |  |  |  |  |  |
| Third trimester | 186 / 214 (86.9%) | 174 / 210 (82.9%) | 0.98 (0.80, 1.21)¶ |  | 1.05 (0.86, 1.28)¶ |  |
| Postpartum | 69 / 94 (73.4%) | 65 / 101 (64.4%) | 0.57 (0.31, 1.06) | 1.83 (1.20, 2.81)† | 0.36 (0.16, 0.84)† | 1.36 (0.92, 1.99) |
| COVID vaccinated |  |  |  |  |  |  |
| Yes | 20 / 21 (95.2%) | 17 / 20 (85.0%) | 1.27 (0.65, 2.45)¶ |  | 1.31 (0.53, 3.23)¶ |  |
| No | 361 / 424 (85.1%) | 338 / 425 (79.5%) | 0.68 (0.51, 0.91)† | 1.28 (1.07, 1.52)† | 0.55 (0.38, 0.81)† | 1.19 (1.01, 1.40)† |

*Abbreviations: CI, confidence interval; HR, hazard ratio.*

*¶ Proportional hazards assumption satisfied and so hazard ratio applies to entire follow-up period.*

*†: 95% confidence interval does not include 1.*

*HR >1 indicates hazard (i.e. instantaneous rate) of outcome was higher in oseltamivir group versus matched control group.*

**Table F6**. Comparison of disease status on days 1, 3, 7, 14 and 28 after index date for oseltamivir recipients and matched controls, overall

|  | **In hospital not ICU** | | **In ICU** | | **Died in hospital** | | **Discharged alive** | |
| --- | --- | --- | --- | --- | --- | --- | --- | --- |
| **Days** | **Oseltamivir** | **Control** | **Oseltamivir** | **Control** | **Oseltamivir** | **Control** | **Oseltamivir** | **Control** |
| 1 | 76.6% | 73.7% | 18.0% | 18.4% | 0.4% | 0.7% | 4.9% | 7.2% |
| 3 | 63.2% | 53.0% | 16.2% | 18.7% | 1.3% | 1.3% | 19.3% | 27.0% |
| 7 | 31.9% | 29.2% | 12.4% | 14.8% | 3.4% | 2.9% | 52.4% | 53.0% |
| 14 | 12.6% | 13.3% | 6.7% | 10.1% | 5.8% | 6.1% | 74.8% | 70.6% |
| 28 | 7.6% | 9.2% | 2.7% | 5.4% | 7.6% | 10.1% | 82.0% | 75.3% |

*Abbreviations: ICU, intensive care unit.*

**Table F7. Baseline** characteristics of oseltamivir recipients and matched controls in the matched cohort, in the subgroup of patients admitted in 2020 (n= 718) and the subgroup admitted from 2021 onwards (n=172)

|  | **Subgroup patients admitted 2020** | | **Subgroup patients admitted 2021 onwards** | |
| --- | --- | --- | --- | --- |
| **Characteristics** | **Oseltamivir**  **recipients**  **(n = 358)** | **Matched controls**  **(n= 360 )** | **Oseltamivir**  **recipients**  **(n=87)** | **Matched controls**  **(n=85)** |
| Age, y (median) [IQR] | 30.2 (25.0, 35.4) | 29.4 (23.6, 34.3) | 27.5 (23.4, 32.2) | 29.4 (23.9, 33.4) |
| Public hospital, % | 196 (54.7%) | 211 (58.6%) | 60 (69.0%) | 51 (60.0%) |
| Pregnant or postpartum |  |  |  |  |
| First trimester | 29 (8.1%) | 26 (7.2%) | 6 (6.9%) | 6 (7.1%) |
| Second trimester | 84 (23.5%) | 81 (22.5%) | 18 (20.7%) | 21 (24.7%) |
| Third trimester | 171 (47.8%) | 168 (46.7%) | 43 (49.4%) | 42 (49.4%) |
| Postpartum | 74 (20.7%) | 85 (23.6%) | 20 (23.0%) | 16 (18.8%) |
| Region of Brazil |  |  |  |  |
| Centre West | 36 (10.1%) | 31 (8.6%) | 7 (8.0%) | 13 (15.3%) |
| North | 42 (11.7%) | 52 (14.4%) | 21 (24.1%) | 12 (14.1%) |
| Northeast | 66 (18.4%) | 80 (22.2%) | 12 (13.8%) | 7 (8.2%) |
| South | 22 (6.1%) | 16 (4.4%) | 7 (8.0%) | 10 (11.8%) |
| Southeast | 192 (53.6%) | 181 (50.3%) | 40 (46.0%) | 43 (50.6%) |
| Self-reported ethnicity |  |  |  |  |
| African | 19 (5.3%) | 21 (5.8%) | 7 (8.0%) | 5 (5.9%) |
| Asian | 1 (0.3%) | 1 (0.3%) | 1 (1.1%) | 0 (0.0%) |
| Caucasian | 112 (31.3%) | 90 (25.0%) | 24 (27.6%) | 29 (34.1%) |
| Indigenous | 1 (0.3%) | 2 (0.6%) | 0 (0.0%) | 0 (0.0%) |
| Mixed | 169 (47.2%) | 179 (49.7%) | 41 (47.1%) | 44 (51.8%) |
| Missing or not reported | 56 (15.6%) | 67 (18.6%) | 14 (16.1%) | 7 (8.2%) |
| Signs |  |  |  |  |
| SpO2 >94% on admission | 241 (67.3%) | 255 (70.8%) | 52 (59.8%) | 43 (50.6%) |
| Symptoms |  |  |  |  |
| Fever | 243 (67.9%) | 248 (68.9%) | 67 (77.0%) | 54 (63.5%) |
| Cough | 292 (81.6%) | 292 (81.1%) | 69 (79.3%) | 72 (84.7%) |
| Sore throat | 86 (24.0%) | 98 (27.2%) | 26 (29.9%) | 18 (21.2%) |
| Dyspnoea | 220 (61.5%) | 208 (57.8%) | 51 (58.6%) | 59 (69.4%) |
| Respiratory discomfort | 165 (46.1%) | 174 (48.3%) | 46 (52.9%) | 42 (49.4%) |
| Nasal congestion | 40 (11.2%) | 35 (9.7%) | 9 (10.3%) | 10 (11.8%) |
| Loss of smell (anosmia) | 22 (6.1%) | 20 (5.6%) | 12 (13.8%) | 11 (12.9%) |
| Loss of taste (ageusia) | 20 (5.6%) | 16 (4.4%) | 9 (10.3%) | 15 (17.6%) |
| Fatigue | 14 (3.9%) | 20 (5.6%) | 24 (27.6%) | 23 (27.1%) |
| Headache | 52 (14.5%) | 51 (14.2%) | 17 (19.5%) | 17 (20.0%) |
| Diarrhoea | 32 (8.9%) | 29 (8.1%) | 6 (6.9%) | 3 (3.5%) |
| Vomit | 34 (9.5%) | 36 (10.0%) | 9 (10.3%) | 13 (15.3%) |
| Abdominal pain | 4 (1.1%) | 9 (2.5%) | 10 (11.5%) | 4 (4.7%) |
| Nausea | 3 (0.8%) | 5 (1.4%) | 1 (1.1%) | 0 (0.0%) |
| Comorbidities |  |  |  |  |
| Cardiovascular disease | 34 (9.5%) | 29 (8.1%) | 3 (3.4%) | 4 (4.7%) |
| Diabetes | 35 (9.8%) | 27 (7.5%) | 3 (3.4%) | 4 (4.7%) |
| Obesity | 19 (5.3%) | 17 (4.7%) | 7 (8.0%) | 8 (9.4%) |
| Asthma | 17 (4.7%) | 16 (4.4%) | 3 (3.4%) | 3 (3.5%) |
| Immunocompromised | 5 (1.4%) | 8 (2.2%) | 1 (1.1%) | 1 (1.2%) |
| Liver disease | 1 (0.3%) | 0 (0.0%) | 0 (0.0%) | 0 (0.0%) |
| Neurological disease | 6 (1.7%) | 4 (1.1%) | 0 (0.0%) | 1 (1.2%) |
| Renal disease | 0 (0.0%) | 2 (0.6%) | 1 (1.1%) | 0 (0.0%) |
| Down syndrome | 0 (0.0%) | 0 (0.0%) | 0 (0.0%) | 0 (0.0%) |
| Vaccinations |  |  |  |  |
| SARS-CoV-2 | 0 (0.0%) | 0 (0.0%) | 21 (24.1%) | 20 (23.5%) |
| Influenza | 82 (22.9%) | 64 (17.8%) | 14 (16.1%) | 14 (16.5%) |
| Days from symptom onset to hospital admission |  |  |  |  |
| 0 | 40 (11.2%) | 35 (9.7%) | 5 (5.7%) | 7 (8.2%) |
| 1 | 48 (13.4%) | 50 (13.9%) | 8 (9.2%) | 14 (16.5%) |
| 2 | 46 (12.8%) | 51 (14.2%) | 15 (17.2%) | 7 (8.2%) |
| 3 | 77 (21.5%) | 73 (20.3%) | 20 (23.0%) | 20 (23.5%) |
| 4 | 60 (16.8%) | 64 (17.8%) | 15 (17.2%) | 15 (17.6%) |
| 5 | 49 (13.7%) | 52 (14.4%) | 15 (17.2%) | 9 (10.6%) |
| 6 | 38 (10.6%) | 35 (9.7%) | 9 (10.3%) | 13 (15.3%) |

**Absolute standardised mean difference >0.10, indicating imbalance in baseline covariates.*

*Abbreviations: IQR, interquartile range; SpO2, peripheral oxygen saturation.*

**Supplementary G**. Sensitivity analysis – patients admitted <14 days from symptom onset

For sensitivity analyses, we rematched baseline covariates and constructed new propensity score models.


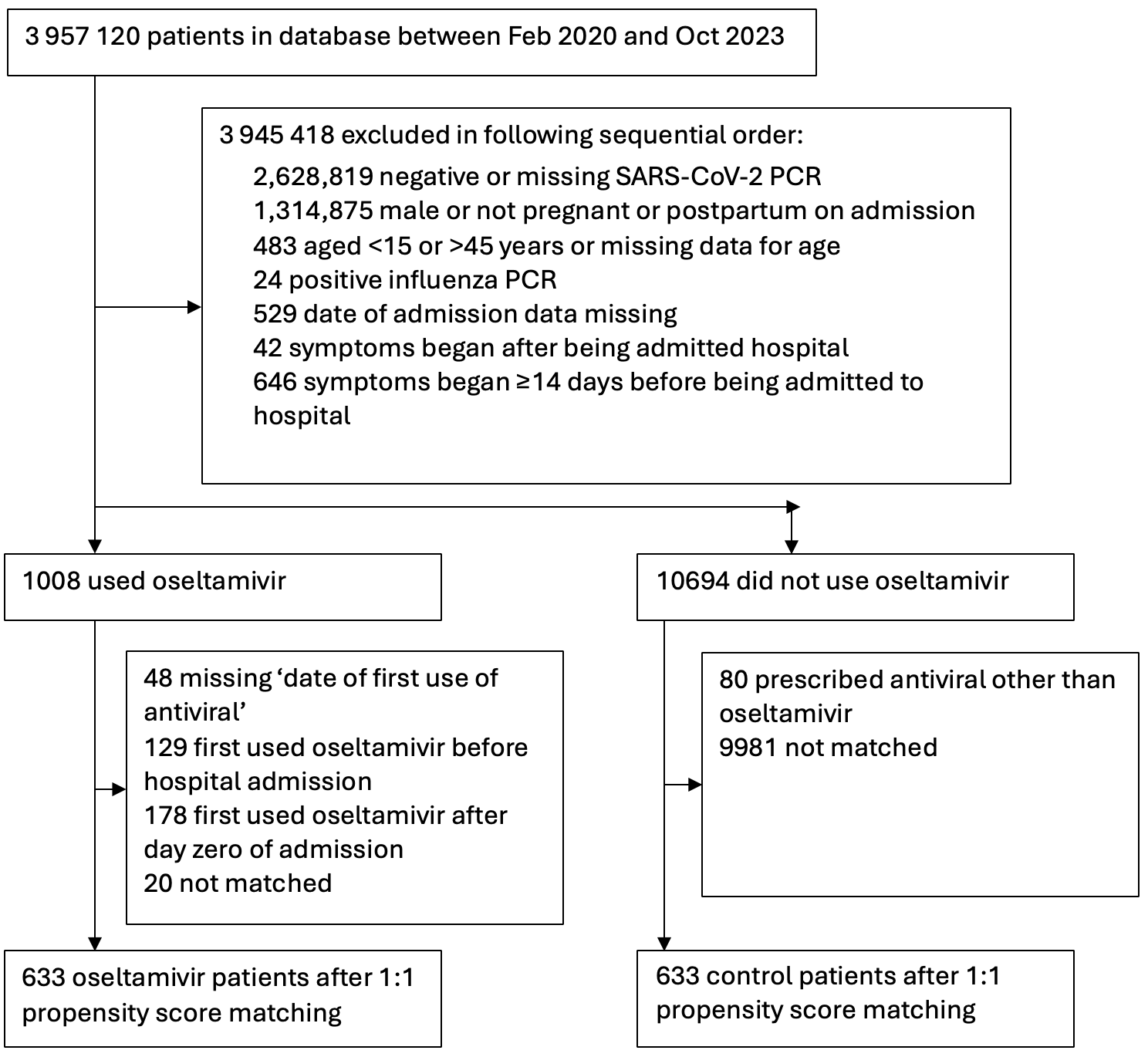
**Figure G1**. Identification of oseltamivir recipients and their matched controls, (sensitivity analyses for patients admitted ≤ 14 days symptom onset)

**Table G1.** Baseline characteristics of oseltamivir recipients and control groups, before and after matching, (sensitivity analyses for patients admitted ≤ 14 days symptom onset)

|  | **Before 1:1 propensity score matching** | | | **After 1:1 propensity score matching** | | |
| --- | --- | --- | --- | --- | --- | --- |
|  | **Oseltamivir**  **(n= 653)** | **Control**  **(n= 10614)** | **Absolute standardised mean difference** | **Oseltamivir recipient**  **(n=633)** | **Control**  **(n=633)** | **Absolute standardised mean difference** |
| Age, y (median) [IQR] | 30.2 [25.2, 35.2] | 30.4 [25.3, 35.4] | 0.04 | 30.3 [25.2, 35.2] | 30.3 [25.5, 35.1] | 0.06 |
| Public hospital, % | 384 (58.9%) | 4799 (45.2%) | 0.28* | 364 (57.5%) | 369 (58.3%) | 0.02 |
| Pregnant or postpartum |  |  |  |  |  |  |
| First trimester | 46 (7.0%) | 761 (7.2%) | <0.01 | 45 (7.1%) | 49 (7.7%) | 0.02 |
| Second trimester | 154 (23.6%) | 2122 (20.0%) | 0.08 | 151 (23.9%) | 135 (21.3%) | 0.06 |
| Third trimester | 314 (48.1%) | 5118 (48.2%) | <0.01 | 303 (47.9%) | 310 (49.0%) | 0.02 |
| Postpartum | 139 (21.3%) | 2613 (24.6%) | 0.08 | 134 (21.2%) | 139 (22.0%) | 0.02 |
| Region of Brazil |  |  |  |  |  |  |
| Centre West | 64 (9.8%) | 1364 (12.9%) | 0.10 | 64 (10.1%) | 55 (8.7%) | 0.05 |
| North | 123 (18.8%) | 450 (4.2%) | 0.37* | 103 (16.3%) | 100 (15.8%) | 0.01 |
| Northeast | 98 (15.0%) | 1739 (16.4%) | 0.04 | 98 (15.5%) | 111 (17.5%) | 0.06 |
| South | 45 (6.9%) | 2317 (21.8%) | 0.59* | 45 (7.1%) | 33 (5.2%) | 0.07 |
| Southeast | 323 (49.5%) | 4744 (44.7%) | 0.10 | 323 (51.0%) | 334 (52.8%) | 0.03 |
| Self-reported ethnicity |  |  |  |  |  |  |
| African | 38 (5.8%) | 570 (5.4%) | 0.02 | 36 (5.7%) | 36 (5.7%) | 0.00 |
| Asian | 3 (0.5%) | 83 (0.8%) | 0.05 | 3 (0.5%) | 1 (0.2%) | 0.05 |
| Caucasian | 204 (31.2%) | 4517 (42.6%) | 0.24* | 204 (32.2%) | 200 (31.6%) | 0.01 |
| Indigenous | 1 (0.2%) | 28 (0.3%) | 0.03 | 1 (0.2%) | 1 (0.2%) | 0.00 |
| Mixed | 307 (47.0%) | 3825 (36.0%) | 0.22 | 291 (46.0%) | 279 (44.1%) | 0.04 |
| Missing or not reported | 100 (15.3%) | 1591 (15.0%) | <0.01 | 98 (15.5%) | 116 (18.3%) | 0.08 |
| Signs |  |  |  |  |  |  |
| SpO2 >94% on admission | 413 (63.2%) | 6778 (63.8%) | 0.01 | 398 (62.9%) | 408 (64.5%) | 0.03 |
| Symptoms |  |  |  |  |  |  |
| Fever | 462 (70.8%) | 5423 (51.1%) | 0.43* | 443 (70.0%) | 425 (67.1%) | 0.06 |
| Cough | 540 (82.7%) | 7083 (66.7%) | 0.42* | 520 (82.1%) | 513 (81.0%) | 0.03 |
| Sore throat | 174 (26.6%) | 2168 (20.4%) | 0.14* | 163 (25.8%) | 150 (23.7%) | 0.05 |
| Dyspnoea | 420 (64.3%) | 5539 (52.2%) | 0.25* | 400 (63.2%) | 381 (60.2%) | 0.06 |
| Respiratory discomfort | 340 (52.1%) | 4382 (41.3%) | 0.22* | 323 (51.0%) | 325 (51.3%) | 0.01 |
| Nasal congestion | 74 (11.3%) | 1240 (11.7%) | 0.01 | 74 (11.7%) | 64 (10.1%) | 0.05 |
| Loss of smell (anosmia) | 64 (9.8%) | 1255 (11.8%) | 0.07 | 64 (10.1%) | 60 (9.5%) | 0.02 |
| Loss of taste (ageusia) | 59 (9.0%) | 1155 (10.9%) | 0.06 | 59 (9.3%) | 49 (7.7%) | 0.05 |
| Fatigue | 71 (10.9%) | 2021 (19.0%) | 0.26* | 71 (11.2%) | 70 (11.1%) | <0.01 |
| Headache | 103 (15.8%) | 1562 (14.7%) | 0.03 | 103 (16.3%) | 92 (14.5%) | 0.05 |
| Diarrhoea | 62 (9.5%) | 864 (8.1%) | 0.05 | 61 (9.6%) | 48 (7.6%) | 0.01 |
| Vomit | 71 (10.9%) | 956 (9.0%) | 0.06 | 69 (10.9%) | 66 (10.4%) | 0.02 |
| Abdominal pain | 21 (3.2%) | 677 (6.4%) | 0.18* | 21 (3.3%) | 19 (3.0%) | 0.02 |
| Nausea | 10 (1.5%) | 144 (1.4%) | 0.01 | 10 (1.6%) | 7 (1.1%) | 0.04 |
| Comorbidities |  |  |  |  |  |  |
| Cardiovascular disease | 53 (8.1%) | 618 (5.8%) | 0.08 | 52 (8.2%) | 47 (7.4%) | 0.03 |
| Diabetes | 47 (7.2%) | 744 (7.0%) | <0.01 | 47 (7.4%) | 37 (5.8%) | 0.06 |
| Obesity | 42 (6.4%) | 674 (6.4%) | <0.01 | 41 (6.5%) | 36 (5.7%) | 0.03 |
| Asthma | 31 (4.7%) | 359 (3.4%) | 0.06 | 31 (4.9%) | 21 (3.3% ) | 0.03 |
| Immunocompromised | 10 (1.5%) | 94 (0.9%) | 0.05 | 9 (1.4%) | 15 (2.4%) | 0.02 |
| Liver disease | 1 (0.2%) | 29 (0.3%) | 0.03 | 1 (0.2%) | 3 (0.5%) | 0.08 |
| Neurological disease | 9 (1.4%) | 67 (0.6%) | 0.06 | 9 (1.4%) | 7 (1.1%) | 0.03 |
| Renal disease | 2 (0.3%) | 58 (0.5%) | 0.04 | 2 (0.3%) | 2 (0.3%) | 0.00 |
| Down syndrome | 0 (0.0%) | 13 (0.1%) | 0.04 | 0 (0.0%) | 0 (0.0%) | 0.00 |
| Vaccinations |  |  |  |  |  |  |
| SARS-CoV-2 | 25 (3.8%) | 1989 (18.7%) | 0.77* | 25 (3.9%) | 26 (4.1%) | <0.01 |
| Influenza | 128 (19.6%) | 1544 (14.5%) | 0.13* | 125 (19.7%) | 116 (18.3%) | 0.04 |
| Days from symptom onset to hospital admission |  |  |  |  |  |  |
| 0 | 45 (6.9%) | 1491 (14.0%) | 0.28* | 45 (7.1%) | 46 (7.3%) | 0.01 |
| 1 | 56 (8.6%) | 878 (8.3%) | 0.01 | 55 (8.7%) | 58 (9.2%) | 0.02 |
| 2 | 64 (9.8%) | 830 (7.8%) | 0.07 | 62 (9.8%) | 61 (9.6%) | <0.01 |
| 3 | 107 (16.4%) | 958 (9.0%) | 0.20* | 99 (15.6%) | 110 (17.4%) | 0.05 |
| 4 | 78 (11.9%) | 838 (7.9%) | 0.12* | 75 (11.8%) | 73 (11.5%) | 0.01 |
| 5 | 66 (10.1%) | 768 (7.2%) | 0.10 | 63 (10.0%) | 65 (10.3%) | 0.01 |
| 6 | 47 (7.2%) | 832 (7.8%) | 0.02 | 47 (7.4%) | 49 (7.7%) | 0.01 |
| 7 | 73 (11.2%) | 1010 (9.5%) | 0.05 | 72 (11.4%) | 69 (10.9%) | 0.01 |
| 8 | 34 (5.2%) | 783 (7.4%) | 0.10 | 33 (5.2%) | 31 (4.8%) | 0.01 |
| 9 | 31 (4.7%) | 682 (6.4%) | 0.08 | 30 (4.7%) | 24 (3.8%) | 0.04 |
| 10 | 26 (4.0%) | 626 (5.9%) | 0.10 | 26 (4.1%) | 26 (4.1%) | 0.00 |
| 11 | 6 (0.9%) | 399 (3.8%) | 0.30* | 6 (0.9%) | 5 (0.8%) | 0.02 |
| 12 | 9 (1.4%) | 317 (3.0%) | 0.14* | 9 (1.4%) | 7 (1.1%) | 0.03 |
| 13 | 11 (1.7%) | 202 (1.9%) | 0.02 | 11 (1.7%) | 9 (1.4%) | 0.02 |
| Quarter of admission |  |  |  |  |  |  |
| Jan-Mar 2020 | 7 (1.1%) | 33 (0.3%) | 0.07 | 7 (1.1%) | 10 (1.6%) | 0.05 |
| Apr-Jun 2020 | 331 (50.7%) | 1242 (11.7%) | 0.78* | 311 (49.1%) | 318 (50.2%) | 0.02 |
| Jul-Sep 2020 | 128 (19.6%) | 980 (9.2%) | 0.26* | 128 (20.2%) | 123 (19.4%) | 0.02 |
| Oct-Dec 2020 | 45 (6.9%) | 727 (6.8%) | <0.01 | 45 (7.1%) | 49 (7.7%) | 0.02 |
| Jan-Mar 2021 | 66 (10.1%) | 2064 (19.4%) | 0.31* | 66 (10.4%) | 68 (10.7%) | 0.01 |
| Apr-Jun 2021 | 37 (5.7%) | 2766 (26.1%) | 0.88* | 37 (5.8%) | 25 (3.9%) | 0.08 |
| Jul-Sep 2021 | 13 (2.0%) | 877 (8.3%) | 0.45* | 13 (2.1%) | 17 (2.7%) | 0.04 |
| Oct-Dec 2021 | 3 (0.5%) | 223 (2.1%) | 0.24* | 3 (0.5%) | 3 (0.5%) | 0.00 |
| Jan-Mar 2022 | 18 (2.8%) | 1018 (9.6%) | 0.42* | 18 (2.8%) | 15 (2.4%) | 0.03 |
| Apr-Jun 2022 | 1 (0.2%) | 255 (2.4%) | 0.57* | 1 (0.2%) | 0 (0.0%) | 0.04 |
| Jul-Sep 2022 | 1 (0.2%) | 117 (1.1%) | 0.24* | 1 (0.2%) | 1 (0.0%) | 0.00 |
| Oct-Dec 2022 | 0 (0.0%) | 180 (1.7%) | 0.14* | 0 (0.0%) | 0 (0.0%) | 0.00 |
| Jan-Mar 2023 | 1 (0.2%) | 74 (0.7%) | 0.14* | 1 (0.2%) | 3 (0.5%) | 0.08 |
| Apr-Jun 2023 | 2 (0.3%) | 50 (0.5%) | 0.03 | 1 (0.2%) | 3 (0.5%) | 0.03 |
| Jul-Sep 2023 | 0 (0.0%) | 8 (0.1%) | 0.03 | 0 (0.0%) | 0 (0.0%) | 0.00 |

**Absolute standardised mean difference >0.10, indicating imbalance in baseline covariates.*

*Abbreviations: SpO2, peripheral oxygen saturation; IQR, interquartile range*

**Figure G2**. Distribution of propensity scores after matching, (sensitivity analyses for patients admitted ≤ 14 days symptom onset)

**Table G2**. Missing data for outcomes after matching, (sensitivity analyses for patients admitted ≤ 14 days symptom onset)

|  | Oseltamivir (n= 633) | Control (n= 633) |
| --- | --- | --- |
|  | n (%) | n (%) |
| Clinical outcome not available or missing* | 32 (5.1%) | 38 (6.0%) |
| Clinical outcome ‘discharged’ but missing date of discharge** | 8 (1.3%) | 22 (3.5%) |

*Note: all of those who had missing data for missing outcome also did not have data for date of clinical endpoint

**Follow-up time was imputed from median times to discharge from those in the same time period, treatment group and symptom onset category

**Figure G3**. Schoenfeld residuals for in-hospital death (all-causes) for oseltamivir recipients and their matched controls

**Figure G4.** Schoenfeld residuals for composite outcome (death or ICU admission, whichever occurred firstly) for oseltamivir recipients and their matched controls

**Figure G5.** Schoenfeld residuals for hospital discharge (alive) for oseltamivir recipients and their matched controls

**Figure G6**. Cumulative incidence function for **in-hospital death** (all-causes) for oseltamivir recipients and their matched controls, (sensitivity analyses for patients admitted ≤ 14 days symptom onset)

**Figure G7**. Cumulative incidence function for **composite outcome** (death or ICU admission, whichever occurred firstly) for oseltamivir recipients and their matched controls, (sensitivity analyses for patients admitted ≤ 14 days symptom onset)

**Figure G8**. Cumulative incidence function for **hospital discharge** (alive) for oseltamivir recipients and their matched controls, (sensitivity analyses for patients admitted ≤ 14 days symptom onset)

**Table G3.** Hazard ratios for **in-hospital death** (all-causes) over entire follow-up for oseltamivir recipients versus their matched controls, overall and by subgroups, (sensitivity analyses for patients admitted ≤ 14 days symptom onset)

|  | **Oseltamivir recipients (n=633)** | **Matched controls (n=633)** | **Oseltamivir recipients versus matched controls** | | | |
| --- | --- | --- | --- | --- | --- | --- |
| **In-hospital death** | **Events (%)** | **Events (%)** | **ARR** | **NNT** | **Cox: cause-specific HR (95% CI)** | **p** |
| Overall | 63/633 (10.0%) | 66/633 (10.4%) | 0.4% | 250 | 0.99 (0.71, 1.38) | 0.95 |
| Time admission |  |  |  |  |  |  |
| <7 days symptom onset | 39/446 (8.7%) | 54/462 (11.7%) | 3.0% | 34 | 0.78 (0.53, 1.17) | 0.23 |
| 7-14 days symptom onset | 24/187 (12.8%) | 12/171 (7.0%) | -5.8% | n/a | 1.80 (0.92, 3.54) | 0.09 |
| Admission date |  |  |  |  |  |  |
| Before 1 Jan 2021 | 37/491 (7.5%) | 48/500 (9.6%) | 2.1% | 48 | 0.77 (0.51, 1.17) | 0.22 |
| On or after 1 Jan 2021 | 26/142 (18.3%) | 18/133 (13.5%) | -4.8% | n/a | 1.64 (0.91, 2.97) | 0.10 |
| SpO2 >94% on admission |  |  |  |  |  |  |
| Yes | 9/398 (2.3%) | 25/408 (6.1%) | 3.8% | 27 | 0.36 (0.17, 0.77) | <0.01 |
| No | 54/235 (23.0%) | 41/225 (18.2%) | -4.8% | n/a | 1.45 (0.98, 2.16) | 0.06 |
| Pregnant or postpartum |  |  |  |  |  |  |
| Third trimester | 23/303 (7.6%) | 28/310 (9.0%) | 1.4% | 72 | 0.79 (0.46, 1.37) | 0.41 |
| Postpartum | 29/134 (21.6%) | 25/139 (18.0%) | -3.6% | n/a | 1.20 (0.70, 2.06) | 0.51 |
| Vaccinated for COVID |  |  |  |  |  |  |
| Yes | 1/25 (4.0%) | 0/26 (0.0%) | -4.0% | n/a | - | - |
| No | 62/608 (10.2%) | 66/607 (10.9%) | 0.7% | 143 | 0.97 (0.70, 1.36) | 0.88 |

**Table G4.** Hazard ratios for **composite outcome** (death or ICU admission, whichever occurred firstly) over entire follow-up for oseltamivir recipients versus their matched controls, overall and by subgroups, (sensitivity analyses for patients admitted ≤ 14 days symptom onset)

|  | **Oseltamivir recipients (n=633)** | **Matched controls (n=633)** | **Oseltamivir recipients versus matched controls** | | | |
| --- | --- | --- | --- | --- | --- | --- |
| **Composite outcome** | **Events (%)** | **Events (%)** | **ARR** | **NNT** | **Cox: cause-specific HR (95% CI)** | **p** |
| Overall | 183/633 (28.9%) | 193/633 (30.5%) | 1.6% | 63 | 0.94 (0.79, 1.10) | 0.42 |
| Time admission |  |  |  |  |  |  |
| <7 days symptom onset | 116/446 (26.0%) | 136/426 (31.9%) | 5.9% | 17 | 0.86 (0.70, 1.07) | 0.17 |
| 7-14 days symptom onset | 67/187 (35.8%) | 57/171 (33.3%) | -2.5% | n/a | 1.08 (0.77, 1.51) | 0.68 |
| Admission date |  |  |  |  |  |  |
| Before 1 Jan 2021 | 131/491 (26.7%) | 145/500 (29.0%) | 2.3% | 44 | 0.90 (0.74, 1.09) | 0.28 |
| On or after 1 Jan 2021 | 52/142 (36.6%) | 48/133 (36.1%) | -0.5% | n/a | 1.05 (0.74, 1.48) | 0.80 |
| SpO2 >94% on admission |  |  |  |  |  |  |
| Yes | 55/398 (13.8%) | 75/408 (18.4%) | 4.6% | 22 | 0.72 (0.52, 0.99) | 0.046 |
| No | 128/235 (54.5%) | 118/225 (52.4%) | -2.1% | n/a | 1.08 (0.86, 1.36) | 0.50 |
| Pregnant or postpartum |  |  |  |  |  |  |
| Third trimester | 75/303 (24.8%) | 80/310 (25.8%) | 1.0% | 100 | 0.95 (0.71, 1.27) | 0.72 |
| Postpartum | 63/134 (47.0%) | 55/139 (39.6%) | -7.4% | n/a | 1.27 (0.91, 1.79) | 0.16 |
| Vaccinated for COVID |  |  |  |  |  |  |
| Yes | 9/25 (36%) | 4/26 (15.4%) | -20.6% | n/a | 2.59 (0.86, 7.83) | 0.09 |
| No | 174/608 (28.6%) | 189/607 (31.1%) | 2.5% | 40 | 0.90 (0.76, 1.06) | 0.22 |

*Abbreviations: ARR, absolute risk reduction; CI, confidence interval; HR, hazard ratio; ICU, intensive care unit; n/a, not applicable; NNT, number needed to treat.*

*Text in bold: p < 0.05.*

*HR >1 indicates hazard (i.e. instantaneous rate) of outcome was higher in oseltamivir group versus matched control group.*

**Table G5.** Hazard ratios for **time-to-hospital discharge** over entire follow-up for oseltamivir recipients versus their matched controls, overall and by subgroups, with split follow-up times, (sensitivity analyses for patients admitted ≤ 14 days symptom onset)

|  | **Oseltamivir recipients (n=633)** | **Matched controls (n=633)** | **Oseltamivir recipients versus matched controls** | |
| --- | --- | --- | --- | --- |
| **Hospital discharge** | **Events (%)** | **Events (%)** | **Cox: cause-specific HR (95% CI)** | |
|  |  |  | Days 0-2 | Days ≥3 |
| Overall | 538/633 (85.0%) | 529/633 (83.6%) | 0.78 (0.62, 1.00) | 1.08 (0.92, 1.24) |
| Time admission |  |  |  |  |
| <7 days symptom onset | 381/446 (85.4%) | 379/462 (82.0%) | 0.72 (0.54, 0.96) | 1.16 (0.98, 1.36) |
| 7-14 days symptom onset | 157/187 (84.0%) | 150/171 (87.7%) | 0.93 (0.75, 1.16) ¶ |  |
| Admission date |  |  |  |  |
| Before 1 Jan 2021 | 427/491 (87.0%) | 426/500 (85.2%) | 0.75 (0.57, 0.98) | 1.08 (0.92, 1.26) |
| On or after 1 Jan 2021 | 111/142 (78.2%) | 103/133 (77.4%) | 1.06 (0.82, 1.38) ¶ |  |
| SpO2 >94% on admission |  |  |  |  |
| Yes | 365/398 (91.7%) | 359/408 (88.0%) | 0.98 (0.85, 1.15) ¶ |  |
| No | 174/235 (74.0%) | 170/225 (75.6%) | 1.05 (0.85, 1.29) ¶ |  |
| Pregnant or postpartum |  |  |  |  |
| Third trimester | 262/303 (86.5%) | 268/310 (86.5%) | 0.95 (0.80, 1.12) ¶ |  |
| Postpartum | 97/134 (72.4%) | 104/139 (74.8%) | 0.68 (0.41, 1.12) | 1.03 (0.73, 1.43) |
| Vaccinated for COVID |  |  |  |  |
| Yes | 23/25 (92.0%) | 24/26 (92.3%) | 0.94 (0.54, 1.68) ¶ |  |
| No | 515/608 (84.7%) | 505/607 (83.2%) | 0.77 (0.60, 0.99) | 1.09 (0.95, 1.25) |

*Abbreviations: CI, confidence interval; HR, hazard ratio.*

*¶ Proportional hazards assumption satisfied and so hazard ratio applies to entire follow-up period.*

*Text in bold: 95% confidence interval does not include 1.*

*HR >1 indicates hazard (i.e. instantaneous rate) of outcome was higher in oseltamivir group versus matched control group.*

**Table G6**. Comparison of disease status on days 1, 3, 7, 14 and 28 after index date for oseltamivir recipients and matched controls, overall*,* (sensitivity analyses for patients admitted ≤ 14 days symptom onset)

|  | **In hospital not ICU** | | **In ICU** | | **Died in hospital** | | **Discharged alive** | |
| --- | --- | --- | --- | --- | --- | --- | --- | --- |
| **Days** | **Oseltamivir** | **Control** | **Oseltamivir** | **Control** | **Oseltamivir** | **Control** | **Oseltamivir** | **Control** |
| 1 | 74.4% | 71.6% | 20.2% | 20.5% | 0.6% | 0.8% | 4.7% | 7.1% |
| 3 | 59.9% | 53.9% | 19.3% | 20.7% | 1.6% | 1.6% | 19.3% | 23.9% |
| 7 | 30.0% | 26.1% | 15.3% | 15.7% | 3.3% | 3.0% | 51.3% | 55.2% |
| 14 | 11.7% | 14.6% | 8.5% | 9.1% | 6.0% | 5.5% | 73.8% | 70.9% |
| 28 | 7.1% | 7.6% | 2.7% | 3.3% | 9.0% | 8.6% | 81.2% | 80.5% |

*Abbreviations: ICU, intensive care unit.*

**Supplementary H.** Dataset and R code

The data and R code used for this project is accessible via OSF Home, under the project: *data and materials for ‘effectiveness of oseltamivir in hospitalised obstetric patients with COVID-19: a retrospective cohort study using a Brazilian national database’*.

<https://osf.io/uykdm/?view_only=308dd2ce1c5745a18ee55a91f247fe21>

**References**

1. Vandenbroucke J, Elm E, Altman D, Gotzsche P, Mulrow C, Pocock S. Strengthening the reporting of observational studies in epidemiology (STROBE): explanation and elaboration. *Ann Intern Med*. 2007;147(8):163-94.

2. Ministry of Health (Brazil). Manual de recomendações para a assistência à gestante e puérpera frente à pandemia de COVID-19. 2020. <https://www.gov.br/saude/pt-br/assuntos/covid-19/publicacoes-tecnicas/guias-e-planos/manual-instrutivo-para-a-assistencia-a-gestante-e-puerpera-frente-a-pandemia-da-covid-19/view> [Accessed 1 Dec 2024].

3. Ministry of Health (Brazil). Manual de recomendações para a assistência à gestante e puérpera frente à pandemia de COVID-19 2˚ edição. 2021. <https://bvsms.saude.gov.br/bvs/publicacoes/manual_assistencia_gestante_puerpera_covid-19_2ed.pdf> [Accessed 1 Dec 2024]

4. Stuart E. Matching methods for causal inference: a review and a look forward. *Stat. Sci*. 2010;25(1):1-21. <https://doi.org/10.1214/09-STS313>

5. Austin P. An introduction to propensity score methods for reducing the effects of confounding in observational studies. *Multivar. Behav. Res*. 2011;46:399–424. <https://doi.org/10.1080/00273171.2011.568786>

6. Zhao Q, Luo J, Su Y, Zhang YJ, Tu G, Luo Z. Propensity score matching with R: conventional methods and new features. *Ann. Transl. Med*. 2021;9(9):812. <https://doi.org/10.21037/atm-20-3998>

7. Wong C, Au I, Lau K, Lau E, Cowling B, Leung G. Real-world effectiveness of early molnupiravir or nirmatrelvir–ritonavir in hospitalised patients with COVID-19 without supplemental oxygen requirement on admission during Hong Kong’s omicron BA.2 wave: a retrospective cohort study. *Lancet Infect. Dis*. 2022;22:1681–93. <https://doi.org/10.1016/S1473-3099(22)00507-2>

8. Wong C, Au I, Lau K, Lau E, Cowling B, Leung G. Real-world effectiveness of molnupiravir and nirmatrelvir plus ritonavir against mortality, hospitalisation, and in-hospital outcomes among community-dwelling, ambulatory patients with confirmed SARS-CoV-2 infection during the omicron wave in Hong Kong: an observational study. *Lancet*. 2022;400:1213-22. <https://doi.org/10.1016/S0140-6736(22)01586-0>

9. Wong C, Lau K, Au I, Xiong X, Lau E, Cowling B. Clinical improvement, outcomes, antiviral activity, and costs associated with early treatment with remdesivir for patients with coronavirus disease 2019 (COVID-19). *Clin. Infect. Dis.* 2022;74(8):1450-8. <https://doi.org/10.1093/cid/ciab631>

10. Zhang Z, Reinikainen J, Adeleke K, Pieterse M, Oudshoorn C. Time-varying covariates and coefficients in Cox regression models. *Ann. Transl. Med*. 2018;6(7). <https://doi.org/10.21037/atm.2018.02.12>

12. Austin P, Fine J. Propensity-score matching with competing risks in survival analysis. *Stat Med.* 2018;38:751-77. <https://doi.org/10.1002/sim.8008>
